# Structural and Functional Insights into Targeting GCCG Sites in the EGFR Promoter by Two DNA Intercalators to Inhibit Breast Cancer Metastasis

**DOI:** 10.1021/acs.jmedchem.4c03203

**Published:** 2025-03-03

**Authors:** Chih-Chun Chang, Hsin-Ju Li, Roshan Satange, Shan-Meng Lin, Tai-Lin Chen, Chi-Chien Lin, Stephen Neidle, Ming-Hon Hou

**Affiliations:** † Graduate Institute of Biotechnology, National Chung Hsing University, Taichung 402, Taiwan; ‡ Graduate Institute of Genomics and Bioinformatics, National Chung Hsing University, Taichung 402, Taiwan; § Post Baccalaureate Medicine, School of Medicine, National Chung Hsing University, Taichung 402, Taiwan; ∥ Institute of Biomedical Science, National Chung Hsing University, Taichung 402, Taiwan; ⊥ The School of Pharmacy, University College London, London WC1N 1AX, U.K.; # Biotechnology Center, National Chung Hsing University, Taichung 402, Taiwan

## Abstract

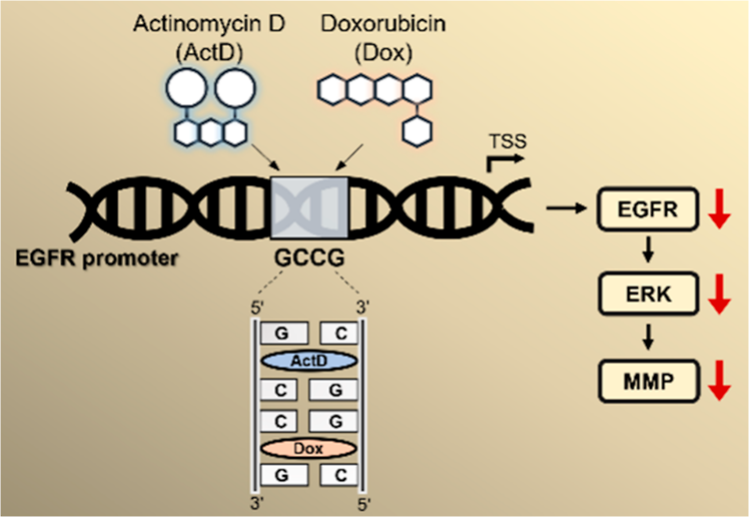

Chemotherapeutic drugs are commonly used to treat cancers lacking targeted therapy options. However, their low specificity limits their treatment effectiveness. We report here that the cooperative binding of doxorubicin (Dox) with actinomycin D (ActD) enhances the specificity for consecutive GCCG sites in DNA. Using X-ray crystallography, we determined the crystal structure of ActD and Dox bound to d(AGCCGT)_2_ DNA. ActD intercalation at the GpC site induces a novel Dox binding mode at the adjacent CpG step. This ensures a snug fit, avoids steric clashes, and enhances the specificity. Transcriptome analysis revealed that combining Dox with ActD synergistically down-regulates EGFR in TNBC cells. Additionally, it reduces EGFR promoter activity. In vivo, the combination significantly suppresses tumor growth and outperforms the standard Dox and cyclophosphamide regimen in inhibiting metastasis. This study highlights targeting the activated EGFR pathway with sequence-specific DNA-targeting drug combinations as a potential TNBC treatment.

## Introduction

Breast cancer is a complex disease with different subtypes that exhibit distinct molecular characteristics and clinical outcomes.^1^ Among these subtypes, triple-negative breast cancer (TNBC) accounts for over 15% of breast cancer cases worldwide.^2^ TNBC tumors lack expression of the estrogen receptor (ER), progesterone receptor (PR), and human epidermal growth factor receptor 2 (HER2), which limits treatment options for later stage disease and makes them a more aggressive and difficult-to-treat subtype. As most targeted and hormonal therapies are less effective in treating TNBC, chemotherapeutic drugs such as anthracyclines, alkylating agents, and platinum-based agents are used.^3^ These drugs can be administered alone, in combination with other chemotherapeutic agents, or as targeted therapy regimens. Although these agents have shown varying success rates, the unresolved issues pertaining to their adverse effects and drug resistance underscore the need for thorough systematic research to develop novel strategies to improve treatment efficacy against TNBC.

**Figure 1:**
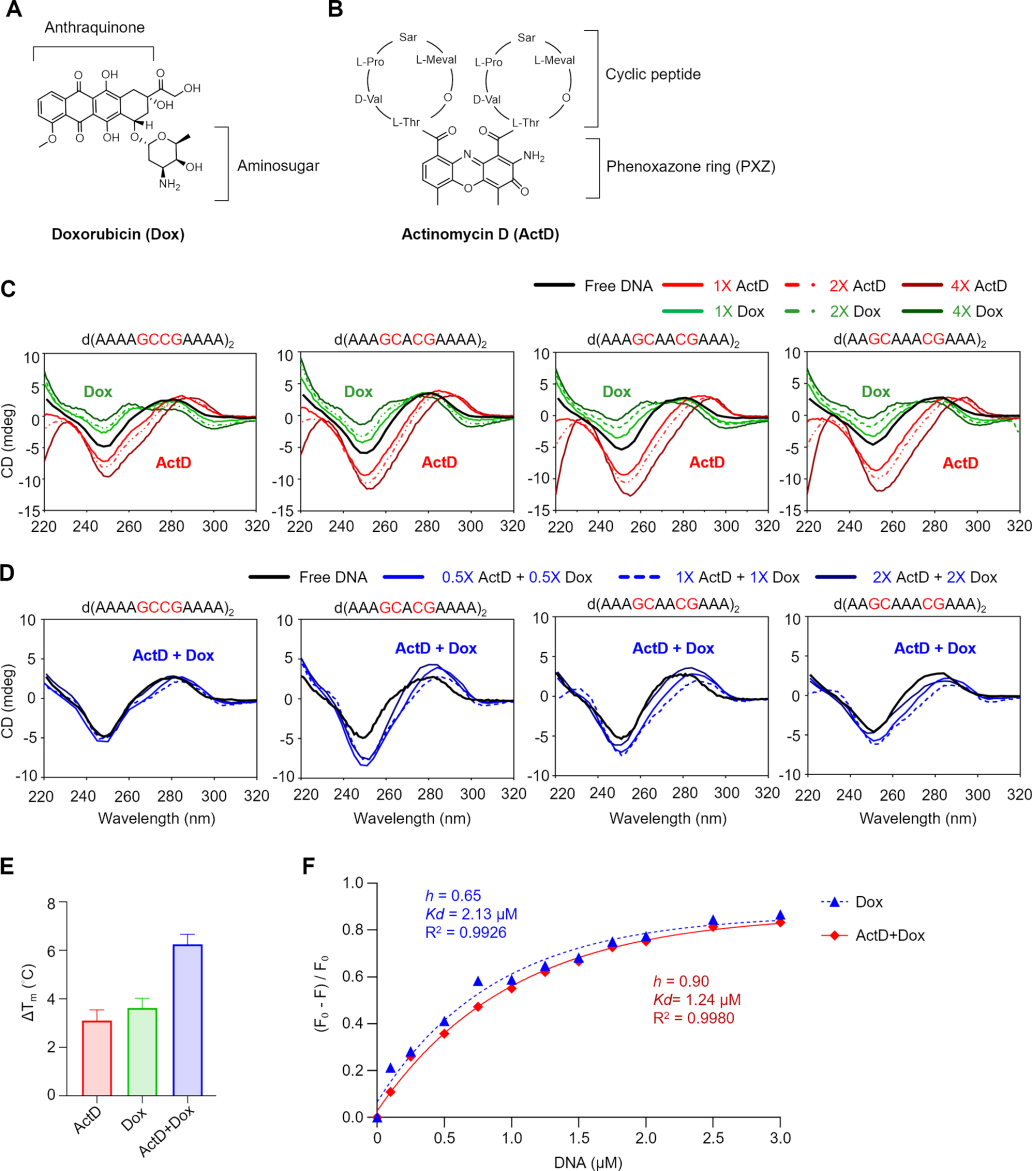
Combination of Dox and ActD enhances the preference for GCCG DNA. Chemical structures of (A) doxorubicin (Dox) and (B) actinomycin D (ActD). (C) Changes observed in the circular dichroism (CD) spectra of various DNA sequences (depicted at the top of each panel) in the presence of ActD alone (red lines), Dox alone (green lines), and (D) combination of ActD and Dox (blue lines). In the figure, 1X, 2X, and 4X represent the molar ratios of the drug to DNA at one, two, and four times, respectively. (E) Change in melting temperature (Δ*T*_m_) of the d(AAAAGCCGAAAA)_2_ duplex induced by ActD, Dox, or a combination, *n* = 3 biological replicates. (F) Nonlinear fitting of fluorescence quenching data using the specific binding model with Hill slope. *K*_d_ is the dissociation constant, and *h* is the Hill coefficient. *h* ≈ 1: noncooperative binding, suggesting independent binding sites; *h* < 1: negative cooperativity, where ligand binding reduces the affinity of additional binding.

Although DNA-targeting agents have proven to be successful for treating TNBC, many of these drugs have limited therapeutic efficacy, dose-dependent toxicity, and life-threatening side effects. For instance, while the anthracycline antibiotic doxorubicin (Dox) (also known as Adriamycin) is widely used to treat breast cancer (Figure 1A) and shows excellent anticancer efficacy, the risk of cardiotoxicity increases with the cumulative dosage of Dox.^4,5^ Typically, the maximum lifetime dose of Dox is around 450–550 mg/m^2^ of body surface area, beyond which the probability of cardiotoxicity increases significantly.^6^ Additionally, Dox is associated with other adverse effects, such as decreased white blood cell production and increased susceptibility to infections.^7,8^ Nonetheless, combination therapy is typically used to improve the efficacy of Dox, and a combination of Dox and cyclophosphamide (the AC regimen) is frequently used to enhance treatment efficacy in invasive breast cancer treatment.^9^ Commonly, many cancer patients are older and may have underlying health vulnerabilities. The use of AC regimens increases the risk of adverse effects, such as myelosuppression and heightened susceptibility to infections.^10^ Thus, the development of novel combination strategies to boost the anticancer effectiveness of Dox while simultaneously reducing its associated toxicity is clinically important.

The possibility of using two different DNA-targeting drugs to treat mismatch repair-deficient cancers has been previously proposed.^11^ This approach also has a broad applicability to other cancer types. Multiple drugs used concurrently can tightly bind to distinct yet specific sites on the genome and induce structural alterations in cancer-cell DNA that may be difficult to repair,^12^ further enhancing therapeutic effects. By generating novel structural changes in cancer-cell genes, we suggest that DNA-targeting drug combination treatments can exhibit synergistic effects and potentially overcome resistance to single-drug therapies. In view of the significant adverse effects associated with Dox treatment, the simultaneous intercalation of Dox with other intercalating drugs may plausibly involve binding to alternative sites. Thus, modulating the sequence specificity of DNA-targeting drugs through the simultaneous use of multiple DNA-targeting agents may help optimize combination chemotherapy for heterogeneous solid tumors, such as TNBC.

The structural basis of Dox-DNA intercalation has been reported previously, where Dox prefers to intercalate at 5′-CpG sites.^13,14^ GC-rich repeat-related sequences are found in the 5′-untranslated regions of certain genes,^15,16^ which are critical for regulating gene expression.^17,18^ In this study, we investigated the mechanism of Dox by combining it with actinomycin D (ActD) (Figure 1B), another potent DNA-intercalating drug.^19^ Our biophysical analyses indicated that the combination of ActD with Dox enhances selectivity to the DNA sequences containing consecutive GCCG sites. The crystal structure of d(AGCCGT)_2_ DNA in complex with ActD and Dox has revealed the basis underlying its enhanced specificity for GCCG sites. Transcriptome analysis further shows that the combination of Dox and ActD significantly downregulates EGFR expression in TNBCs. Notably, ranging from 30 to 70% of TNBC cases show EGFR overexpression,^20,21^ and the EGFR promoter region contains a high frequency of GCCG motifs. The biological effects of this drug combination involve the suppression of EGFR and its related signalling network, which consequently exhibit enhanced antitumor activity and reduced metastasis in TNBC in vivo models, indicating a promising new avenue for optimizing combination chemotherapy regimens.

## Results

### ActD and Dox Combination Shows the Preference for Consecutive GCCG-Site Containing DNA

To understand the binding preferences of ActD, Dox, and their combination, we designed various oligonucleotide duplexes containing their preferred dinucleotide-binding sites, GpC for ActD and CpG for Dox, with 0, 1, 2, and 3 flanking AT base pairs between these sites (Table S1). The CD spectra of these sequences show that all of the duplexes adopted a right-handed conformation. Upon titration of ActD alone, the spectra demonstrated significant concentration-dependent changes, including a red shift from 275 to 290 nm and an increase in the negative peak intensity from 250 to 260 nm in all DNA duplexes, irrespective of the flanking base pairs between the GpC and CpG sites. These spectral changes indicate DNA unwinding upon ActD intercalation.^22^ Similarly, individual treatment with Dox resulted in a major shift of the band at 275 nm into two bands at 260 and 282 nm in a concentration-dependent manner. In addition, there were CD intensity changes at 250 and 260 nm in the Dox-DNA spectra. These spectral shifts indicate a partial B-to-A DNA transition and Dox intercalation in these duplexes (Figure 1C).^23^ Interestingly, when the two drugs were titrated in equimolar ratios, there was no change in the CD spectra of DNA duplexes containing consecutive GCCG sites. On the other hand, in the sequence containing one AT base pair flanked between GpC and CpG sites, the spectra showed a perturbation from 275 to 285 nm with an increase in the CD intensity peak, while the CD intensity at 250 nm decreased significantly (Figure 1D). These observations were consistent with the other two duplexes containing two and three AT base pairs flanked between GpC and CpG sites. These alterations in spectral characteristics suggest that ActD can compete with Dox binding sites when at least one base pair is present between the GpC and CpG sites, allowing it to bind nonspecifically with DNA. Furthermore, under similar conditions, the presence of continuous GCCG sites enables the two drugs to form a stable complex, irrespective of the ActD concentration. The thermostability data corroborated these findings, indicating that the combination of Dox and ActD stabilized the GCCG motif sequence more effectively than the individual drug treatments (Figures 1E and S1A). To investigate whether ActD affects the binding behavior of Dox to the GCCG motif, we performed a fluorescence quenching assay (Figure S1B). The Hill constant for Dox alone was 0.65, suggesting that Dox binds to CpG and nonspecifically to other sites. In the presence of ActD, the Hill constant increased to 0.9, indicating that ActD facilitates the specific binding of Dox to CpG within the GCCG motif. Moreover, the dissociation constant (*K*_d_) for the binding of Dox to the GCCG motif decreased upon the addition of ActD (Figure 1F). These results suggest that the synergy between ActD and Dox enhances the selective binding of Dox to DNA containing the GCCG motif.

### Structural Basis of ActD and Dox Binding to the GCCG-Containing DNA

**Figure 2:**
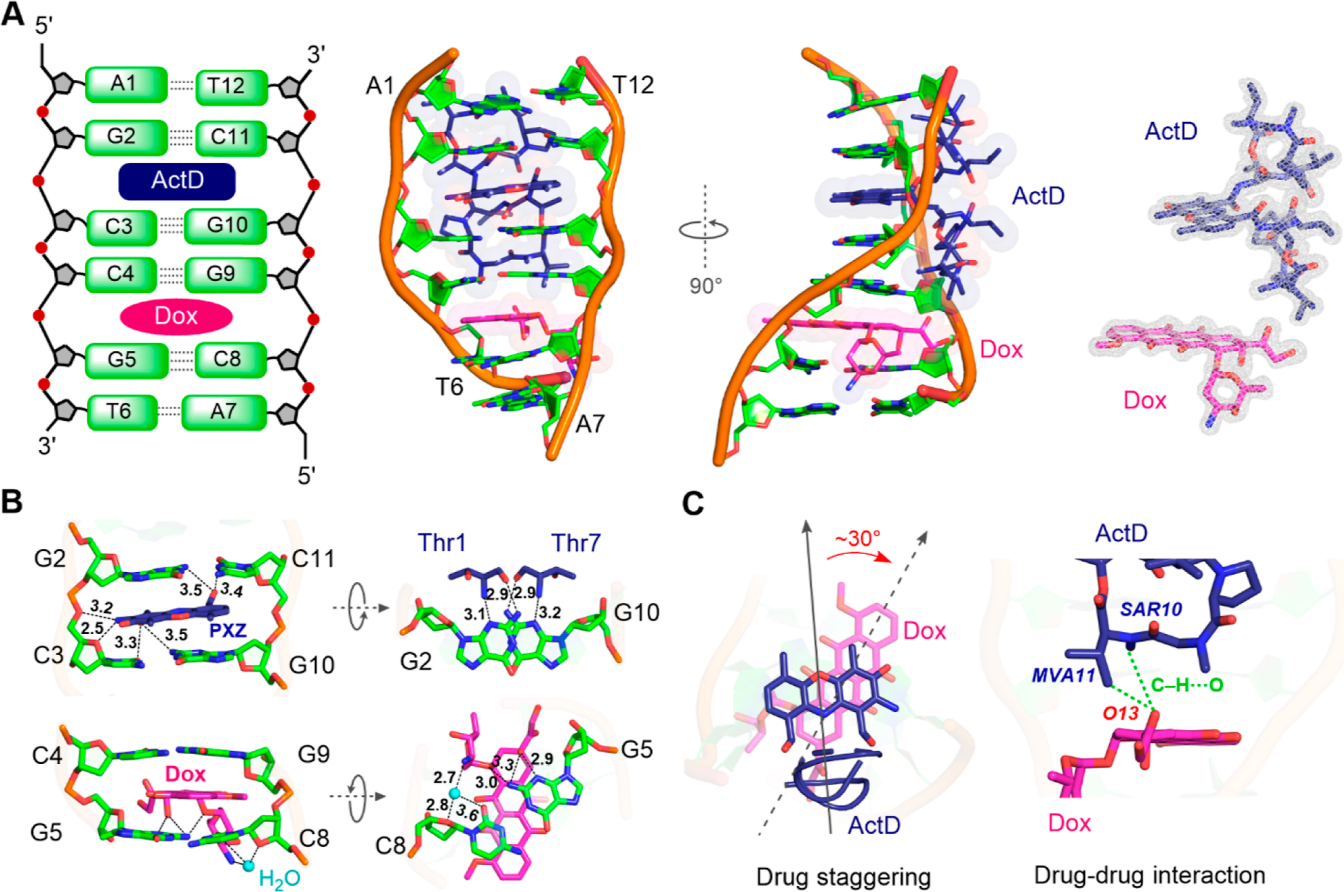
Structural basis of ActD and Dox combination stabilizing GCGC DNA. (A) Schematic representation of the ActD-Dox- d(AGCCGT)2 complex showing ActD binding at 5′-G2pC3 and Dox binding at 5′-C4pG5 steps (PDB ID: 9JL7). The overall structure of the ActD-Dox-DNA complex in an asymmetric unit viewed from side and front views is shown. In the figure, the DNA backbone is represented in an orange cartoon, and base pairs are shown in green sticks. The two drug molecules, ActD and Dox, are represented in blue and pink, respectively. The 2|F_0_| – |F*c*| electron density maps of Dox and ActD (contoured at 1.0 σ) are shown in gray mesh in the right panel. (B) Intercalation and hydrogen bond interactions of ActD and Dox in the crystal structure. Hydrogen bonds are shown by black dashed lines. PXZ: phenoxazone, Thr: threonine, and H_2_O: water molecule. (C) Drug–drug interaction network in the complex. Left: staggering of ActD and Dox molecules by approximately 30° resulted in continuous stacking interactions between drug and DNA base pairs. Right: the C–H···O interactions between carbon atoms of MVA and SAR residues of ActD and O13 of Dox enhance the effects of two drugs bound to the DNA.

To reveal the basis underlying the preference for the GCCG motif shown by the combination of ActD and Dox, we determined the cocrystal structure of a ternary complex in which both drugs are bound to the d(AGCCGT)_2_ DNA (1.57 Å). The bases in the DNA duplex are designated as 1–6 on one strand and 7–12 on the complementary strand (Figure 2A). In the crystal structure, an ActD and a Dox molecule are each intercalated between the G2pC3 and C4pG5 sites in DNA, respectively. The simultaneous intercalation of both drugs induces distinct structural changes in the DNA backbone, including significant unwinding at the ActD-intercalation site. The structure contained three manganese (Mn^2+^) ions, one chloride (Cl-) ion, and 93 water molecules. Notably, two Mn^2+^ ions were associated with the N7 atoms of the G5 and G9 bases, exhibiting an octahedral coordination with five water molecules (Figure S2A). We also observed end-to-end π–π stacking interactions within the symmetry-related duplex that further stabilized the overall crystal structure complex (Figure S2B). All base pairs in the complex structure adopted Watson–Crick base-pairing geometry. Most nucleotides had a glycoside torsion angle (χ) value of approximately −100°, and their sugar puckers were predominantly in the C2′-endo conformation, indicating a close relationship to B-form DNA. Simultaneous intercalation of both drugs induced distinct structural changes in the backbone, including significant unwinding at the ActD-intercalation site. The ActD-binding step (G2C3/G10C11) exhibited higher roll angle (3.7°) values and a lower twist angle (10.5°), resulting in significant backbone unwinding. Conversely, at the Dox-intercalation site (C4G5/C8G9), these values were reversed (roll = −3.8° and twist = 31.5°), indicating overwinding of the backbone (Figure S2C). These variations, coupled with the different staggering of the ActD and Dox chromophores, led to the smooth bending of the backbone near the Dox-binding site, yielding an overall asymmetric structure. Moreover, alterations in base-pair rise and buckle parameters were evident at both the ActD- and Dox-intercalation sites, indicating the distinct effects of each drug on the DNA structure. The base-pair rise at the ActD-intercalation interface (G2C3/G10/C11) was approximately 7.4 Å, while that at the Dox-binding site was 7.0 Å (C4G5/C8G9). This increase in the rise distance may be attributable to the fact that ActD possesses a chromophore (phenoxazone; PXZ), which intercalates in parallel with the base pairs and has a bulky cyclic ring structure. In contrast, the anthraquinone ring of Dox intercalates perpendicular to the base pairs, necessitating fewer structural alterations in the backbone conformation and base morphology. Notably, while most base pairs exhibited positive values for the buckle parameter, the G5-C8 base pair at the Dox-binding site demonstrated a large negative value of −13.4°. These differences at the two drug-intercalation sites further manifested in the distinct orientation of the ActD phenoxazone (PXZ) moiety and the Dox aglycone anthraquinone ring structure.

### New Binding Mode of Dox, Water-Mediated Dox-DNA Interactions, and Drug–Drug C–H···O Interactions Enhance the Specificity of ActD and Dox Combination to GCCG Sites

A detailed analysis of the binding sites of ActD and Dox has revealed how the combination of these drugs enhances the specificity to GCCG sites (Figure 2B). At the ActD-intercalation site, there are extensive stacking and hydrogen-bond interactions between the phenoxazone (PXZ) ring and two threonine (Thr) moieties with guanine bases G2 and G10. At the Dox-intercalation site, Dox has three hydrogen-bonded interactions with the N2/N3 atoms of guanine G5. In addition, a water molecule mediates specific hydrogen-bonded interactions between the amino sugar nitrogen atom of Dox and the oxygen atom of cytosine C8. While earlier structural studies of Dox–DNA complexes have identified water molecules around the major and minor grooves that facilitate interactions between the second or third hydration shells, direct water-mediated interactions between Dox and DNA base pairs have not been previously reported.^13^ These alterations at the Dox-binding site are likely caused by ActD-induced changes in the DNA, which subsequently influence the alignment of DNA bases and allow water molecules to come into proximity and form direct interactions. In comparison with the previously reported ActD–DNA and Dox–DNA complex structures, the ActD-binding site has a close resemblance to the earlier complex,^24^ with a root-mean-square deviation (RMSD) of 0.4 Å (Figure S3A), while the Dox-binding site exhibited significant deviations from the previous Dox–DNA complex structure, with an RMSD of ca. 1.0 Å.^14^ The bulky cyclic peptide ring of ActD widens the minor groove, resulting in the amino sugar moiety of Dox being rotated by ca. 180° in comparison with the previous Dox–DNA crystal structure. This results in an opposite orientation of Dox compared to that observed earlier. Additionally, the present structure shows an increase in the interstrand P–P distances at both drug-binding sites (Figure S3B), with the average P–P distance at the Dox-intercalation site increasing by 0.5 Å from the typical value of 16.6 Å in the previously reported Dox–DNA structure. The changes in the amino sugar orientation, coupled with the increased P–P distance, cause displacement of the G5-C8 base pair (with a negative buckle of −13.4°) from its original position, creating space to accommodate the water molecule near Dox and further facilitating the stabilization process. Furthermore, the staggering of ActD and Dox by approximately 30° relative to the plane of the DNA base pairs, along with the drug–drug interaction network, significantly enhances the binding preference of the ActD and Dox combination at the GCCG sites in DNA (Figure 2C). Additionally, the side chains of ActD and Dox are in close contact, with a C–H···O 3.4 Å interaction between the carbonyl atom of the Dox side chain and a carbon atom of a valine in ActD. This helps to ensure a snug fit, prevents steric clashes between the two drugs, and enhances their specificity for binding to consecutive dinucleotide steps in DNA.

### Transcriptome Analysis Revealed that ActD and Dox Combination Treatment Synergistically Inhibits the EGFR Pathway in TNBC Cells

**Figure 3:**
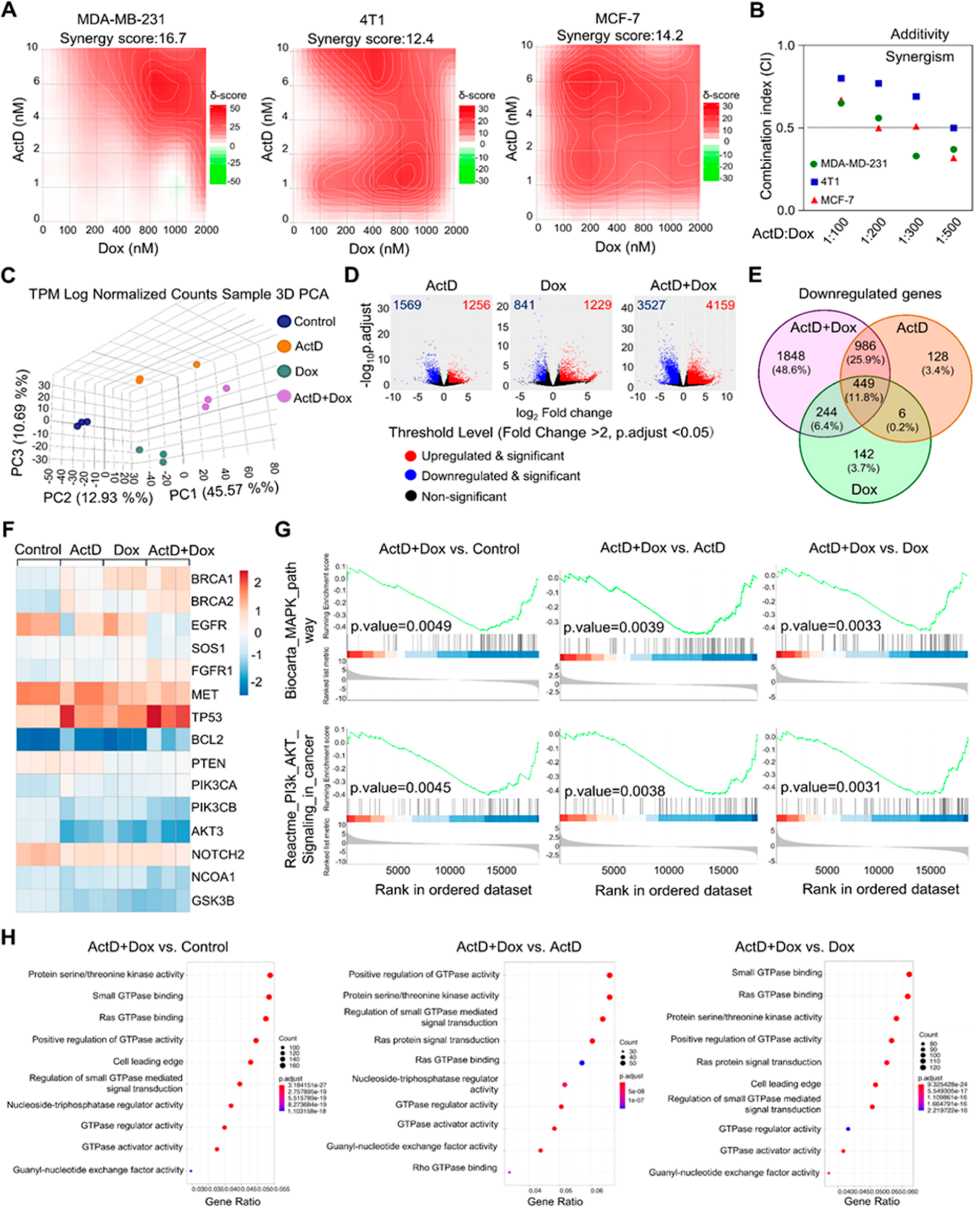
Synergistic effects of ActD and Dox combination in TNBC cells. (A) Synergy distribution and score were estimated using SynergyFinder 3.0 with the zero-interaction potency (ZIP) method (*n* = 3, biological replicates). (B) CI values are determined by different molar ratios of ActD and Dox using CompuSyn 1.0 (mean of three experiments), CI index > 1.1 means antagonism, 1.1 ≥ CI index > 0.9 indicates addictive effect, and CI index ≤ 0.9 indicates synergism (*n* = 3, biological replicates). (C) Principal component analysis (PCA) of the RNA-seq (*n* = 3, biological replicates). (D) Volcano plot showing the differences in gene expression between experimental condition. (E) Venn diagram displaying the overlap and statistically significant downregulated differentially expressed genes. (F) Heat map of differentially expressed genes in genetic markers of TNBC. (G) GSEA indicates enrichment of gene sets in both the MAPK and PI3K pathways. (H) Gene ontology (GO) analysis performed on 1000 downregulated differentially expressed genes resulting from comparisons between combinations with each treatment group.

Our biophysical and structural observations reveal that the combination of ActD and Dox displays a preference for cooperative binding to consecutive GCCG sites in DNA. To assess the relevance of these findings in breast cancer cells, we conducted in vitro experiments using TNBC cells. We used MDA-MB-231 (human TNBC cells), 4T1 (murine TNBC cells), and MCF-7 (human ER+, PR+, and HER2-breast cancer cells) cells to determine the effects of Dox and ActD alone on cell viability and compared them to other commonly used chemotherapy drugs. The IC_50_ of ActD in these cell lines was approximately 100 times lower than those of Dox and the other drugs (Table S2). To examine the synergism of ActD and Dox in these cells, they were treated with combinations of the two drugs, and the effects were evaluated using SynergyFinder and a combination index (CI). The dose–response matrix was arranged in various concentrations of ActD (from 1 to 10 nM) and Dox (from 0.1 nM to 1 μM) to verify cell growth inhibition. The two-drug combination demonstrated significant synergistic effects, with a synergy score greater than 10 (Figure 3A). Second, for the CI analysis, we set up different ratios (nanomolar molar ratio 1:100 to 1:500) of ActD and Dox according to the IC_50_ values. The CI values (0.32–0.8) also showed good synergistic effects for each ratio of the combination group (Figures 3B and S4A and Table S3). Moreover, ActD increased the G2/M arrest induced by Dox in the combination treatment (Figure S4B). These preliminary experiments indicate that the combination treatment synergistically inhibits the viability of TNBC cells.

Next, we conducted transcriptome analysis of MDA-MB-231 cells treated with ActD, Dox, or a combination of both using RNA-seq. The data set contained the expression data for 18154 genes. Principal component analysis showed the gene expression clusters for each treatment (Figure 3C). Differentially expressed gene (DEG) analysis indicated that the combination significantly affected gene expression, causing the upregulation of 4159 genes and downregulation of 3527 genes (Figure 3D). The comparison between the combination and single treatments revealed 1848 downregulated DEGs in the combination group (Figure 3E). First, we evaluated the expression of clinical TNBC marker genes, including those involved in the BRCA1, BRCA2, EGFR, FGFR, P53, PI3K, and NOTCH pathways.^25^ The combination treatment increased the expression of genes related to DNA repair (BRCA1 and BRCA2) and apoptosis (TP53 and BCL2). The expression of P53 was elevated by this combination, consistent with previous observations.^26^ Notably, the expression of EGFR and its downstream PI3K pathway was significantly reduced by the combination treatment (Figure 3F and Table S4). Moreover, protein–protein interaction analysis showed that the combination treatment primarily inhibited the EGFR network (Figure S5). Gene Set Enrichment Analysis (GSEA) for further examination of the downstream gene expression of EGFR illustrated that the combination treatment caused significant downregulation enrichment of both the MAPK and PI3K pathways (Figure 3G).

The DEGs were further analyzed using the Gene Ontology (GO) classification, which demonstrated that the combination treatment mostly downregulated genes related to protein serine/threonine kinase and GTPase activities (Figure 3H). Protein serine/threonine kinases regulate diverse cellular processes and perform critical post-translational modifications through phosphorylation reactions. The 5′-UTRs for protein serine/threonine kinase activity contain CGG trinucleotide repeats (TNRs).^27^ Thus, we suggest that this drug combination predominantly targets DNA sequences containing specific GCCG motifs (complementary to the reverse strands of CGG TNRs). Individual treatments with ActD or Dox were involved in the modulation of GTPase activity, as well as the regulation of cell–cell contacts and cell development, respectively (Figure S6A). In contrast, the combination treatment upregulated 2581 DEGs (Figure S6B), which were associated with DNA-binding transcription activity, DNA packaging, and the cell cycle. High DNA packaging leads to suppression of gene expression^28^ (Figure S6C,D). Overall, the combination of ActD and Dox suppresses the expression of essential genes such as those related to EGFR and protein serine/threonine kinase activity in TNBC cells, due, we suggest, to enhanced binding to DNA at GCCG sites.

### Binding of the ActD and Dox Combination to the EGFR Promoter Downregulates EGFR Expression

**Figure 4:**
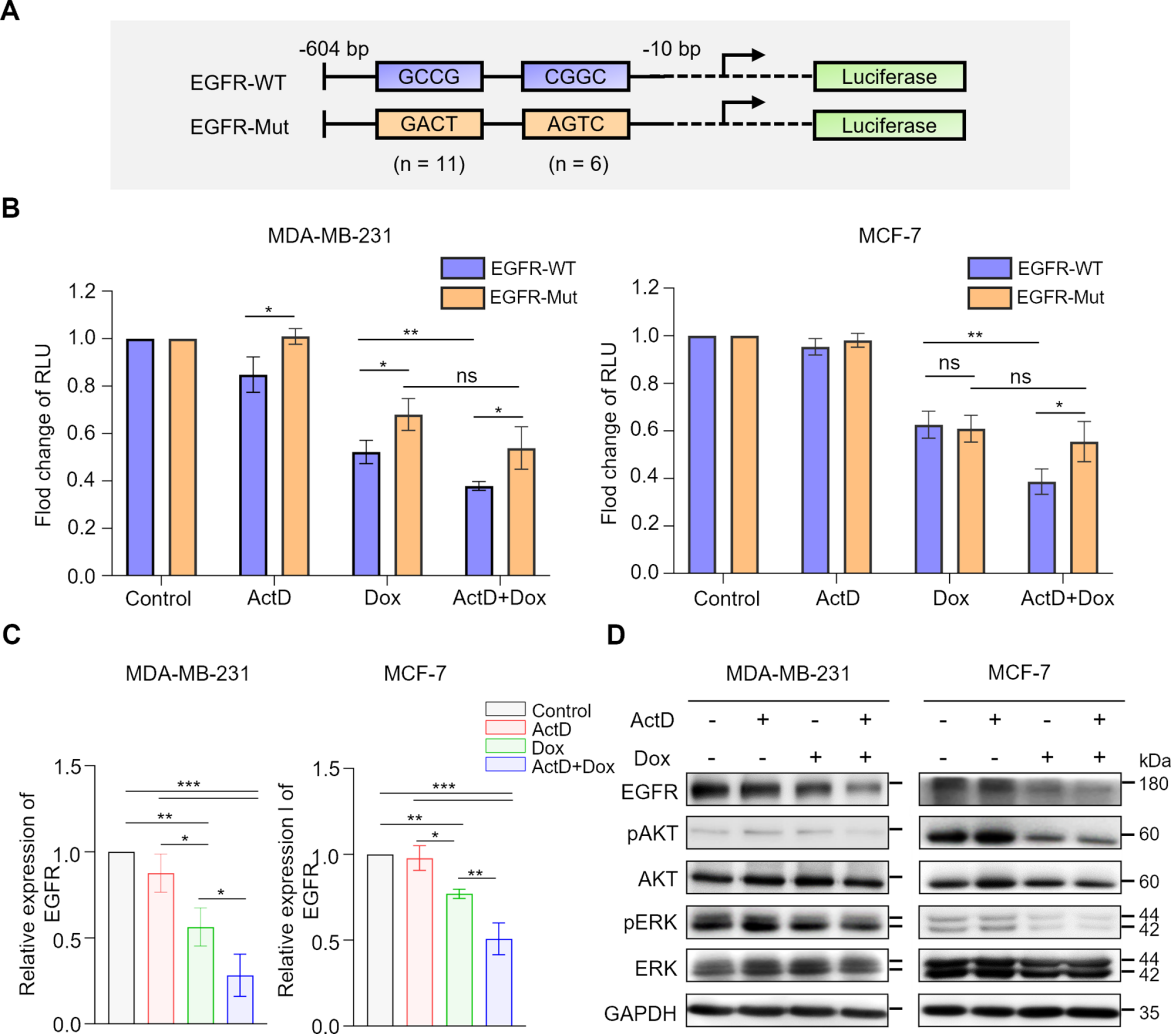
Combination treatment with ActD and Dox modulates EGFR expression in human breast cancer cells. (A) Schematic representation of the EGFR promoter illustrates binding sites for ActD and Dox located at −10 and −604 base pairs upstream of the ATG start codon. Mutations in the EGFR promoter were created using GACT or AGTC motifs to replace GCCG or CGGC, respectively. (B) MDA-MB-231 and MCF-7 cells were treated with 2 nM ActD and 600 nM Dox for 18 h. Luciferase activity is shown as the fold change in relative light units (RLU) compared to the control group. Data are presented as mean ± SD, with *n* = 3 biological replicates. Statistical significance was evaluated using two-tailed *P* values: *P < 0.05, **P < 0.01, and ns = not significant. (C) MDA-MB-231 and MCF-7 cells were treated with 2 nM ActD and 600 nM Dox for 24 and 30 h, respectively. EGFR RNA expression was measured using real-time PCR (qPCR). *P*-values from one-way ANOVA tests comparing each group are indicated as follows: *P < 0.05, **P < 0.01, and ***P < 0.001. (D) Western blot analysis of the EGFR signaling pathway in MDA-MB-231 and MCF-7 cells treated with 2 nM ActD and 600 nM Dox for 30 and 36 h, respectively.

The EGFR promoter and its 5′-untranslated region are characterized by high GC content (∼88%)^29^ and contain a substantial number of GCCG or CGGC motifs (Figure S7). To investigate the potential of ActD and Dox to bind to the EGFR promoter region, we performed luciferase reporter assays in MDA-MB-231 and MCF-7 cells. The promoter region from −604 to −10 bp upstream to the EGFR transcription start site contained 11 GCCG and 6 CGGC sequence motifs. For control experiments, these motifs were mutated to GACT and AGCT, respectively (Figure 4A). Both the wild-type and mutated promoter sequences were cloned into a firefly luciferase reporter vector to assess the effects of ActD, Dox, and their combination on EGFR promoter activity. The results, as shown in Figure 4B, revealed that the combination treatment significantly reduced EGFR promoter activity compared with the individual treatments. This reduction was not observed in the mutated promoter group in both cancer cell lines. We further assessed the expression of EGFR at both the RNA and protein levels following the drug treatments. The combination effectively down-regulated EGFR expression and altered the phosphorylation states of AKT and ERK in MDA-MB-231 and MCF-7 cells (Figure 4C,D). These results confirmed that the ActD and Dox combination suppresses the EGFR signaling pathway by specifically targeting and binding to the EGFR promoter region.

### In Vivo Antitumor Efficacy and Toxicity of ActD and Dox Combination Therapy in the MDA-MB-231 Xenograft Model

**Figure 5:**
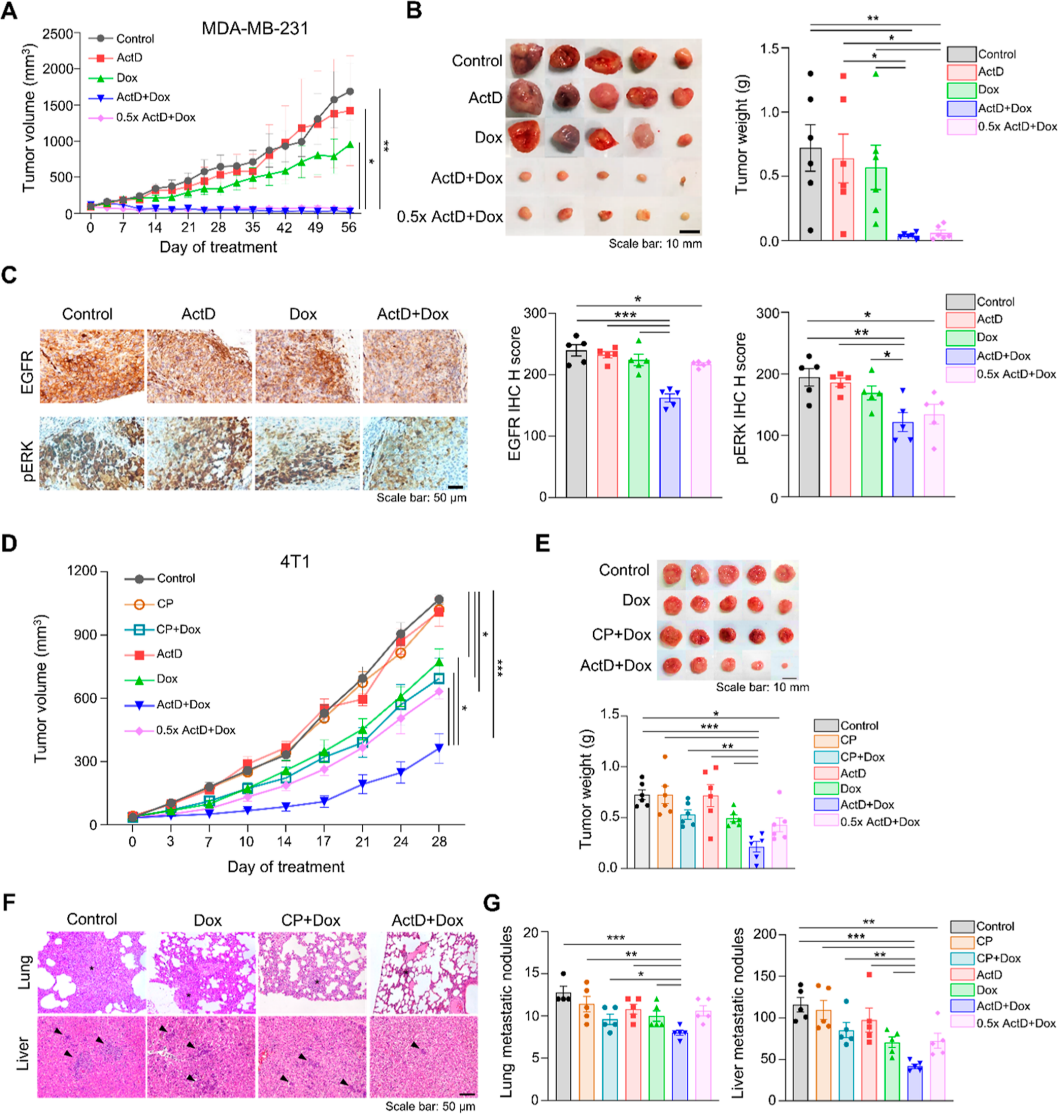
Combination of ActD and Dox suppresses tumor growth in the MDA-MB-231 xenograft and 4T1 orthotropic TNBC model. (A) Tumor growth curves and (B) tumor pictures from MDA-MB-231 xenograft mice treated with control (PBS), ActD, Dox, and combination (*n* = 6 per group). The five horizontal images for each treatment group represent different samples. Scale bars: 10 mm. The weight of the tumors was quantified and compared between the treatment groups and the control group. (C) IHC staining and statistical results of EGFR and pERK on MDA-MB-231 tumor sections (*n* = 5 per group), Scale bar: 50 μm. Data is presented as mean ± SEM (*P < 0.05, **P < 0.01, ***P < 0.001). (D) Therapeutic efficacy of each treatment is assessed by tumor growth curves and (E) average weights of tumors (*n* = 6 per group). Scale bar: 10 mm. (F) Mouse liver sections were evaluated by H&E staining and observed under a light microscope. Asterisks or arrows were used to represent metastatic lesions. (G) Statistic result of lung and liver metastatic nodules (*n* = 4 or 5 per group). The data are presented as mean ± SEM (*P < 0.05, **P < 0.01, and ***P < 0.001). Scale bar: 50 μm.

Studies in animal models have shown that high doses of Dox (cumulative dose, 2–20 mg/kg) lead to weight loss, heart, liver, and kidney damage, and gastrointestinal inflammation in mice of various strains.^30−32^ Therefore, we conducted a preliminary test of dose tolerance in BALB/c nude mice using 3 mg/kg Dox (*n* = 2). Within a week, the mice showed a hunched posture and weight loss (the mice were euthanized when their body weight was reduced by 20%). To avoid systemic toxicity, we combined low doses of ActD and Dox and tested their antitumor activity and drug toxicity. The clinical dosage of ActD was 15 μg/kg daily for 5 days, totaling 75 μg/kg once every 3 weeks.^33,34^ According to the recommended dosage, the mice received 20 μg/kg of ActD and 0.5 mg/kg of Dox (ratio of 1:25) for 8 weeks (cumulative Dox dose, 4 mg/kg). Combination therapy significantly reduced the xenograft tumor volume, even at the half dosage of the combination (Figure 5A,B).

Moreover, these doses of ActD and Dox had no effect on body, liver, or heart weights (Figure S8A). The in vitro RNA-sequencing (RNA-seq) findings indicated that the combination of ActD and Dox decreased the level of EGFR expression. Therefore, we quantified the expression of EGFR, its downstream signaling molecule pERK, and the proliferative marker Ki67 in the MDA-MB-231 xenograft model using immunohistochemical (IHC) staining. Combined treatment with ActD and Dox reduced the expression levels of EGFR, pERK, and Ki67 in the tumor tissues (Figures 5C and S8B). Overall, the combination treatment effectively suppressed MDA-MB-231 xenograft growth with concomitant inhibition of the EGFR signaling pathway and showed no adverse effects in the treated mice.

### Combination Therapy Inhibits Metastasis in the 4T1 Orthotopic Breast Cancer Model

In clinical practice, the AC regimen (adriamycin and cyclophosphamide) is a first-line treatment for invasive breast cancer. However, hematological and gastrointestinal toxicities have been observed in patients receiving AC regimen.^32,35,36^ A synergistic therapeutic effect was observed after the combined administration of metronomic chemotherapy with ActD and Dox. We then compared the anticancer efficacy and hematological toxicity of the AC regimen with those of the ActD–Dox combination using a 4T1 breast tumor-bearing mouse model that mimics the aggressiveness of human breast carcinoma. To evaluate the therapeutic effect, mice were treated with cyclophosphamide (CP) (5 mg/kg), ActD (20 μg/kg), Dox (0.5 mg/kg), or a combination of these treatments for 4 weeks (cumulative Dox dose, 2 mg/kg). The results indicated that the combination of ActD and Dox significantly reduced orthotopic tumor volume and weight (Figure 5D,E). The half dose of the combination shows effects similar to those of the individual Dox treatment. 4T1 tumor-bearing mice exhibit metastases to distant organs, including the liver, spleen, lungs, and intestines. Treatment with ActD and Dox effectively decreased metastatic lung and liver nodules (Figure 5F,G). The results of IHC analysis for the MDA-MB-231 model were consistent, showing reduced expression of EGFR, pERK, and Ki67 following ActD and Dox treatments (Figure S8C,D). Importantly, in the 4T1 tumor-bearing mouse models, the ActD–Dox combined group showed significantly longer survival than the AC-treated group and other single-treatment groups (Figure S8E). For hematological toxicity assessment, we tested high doses (4-fold) of cyclophosphamide (20 mg/kg), ActD (80 μg/kg), Dox (2 mg/kg), or their combinations in nontumor-bearing mice. Blood samples were analyzed by complete blood count (CBC). Hematological analysis revealed that Dox decreased the total number of white blood cells, primarily lymphocytes, but no significant reduction was observed in the half combination treatment group (Figure S8F and Table S5). In conclusion, the combination of ActD and Dox exhibited significant antitumor activity and reduced metastasis in a TNBC orthotopic model.

### ActD and Dox Combination Suppresses the Migration and Invasion of TNBC Cells through the EGFR/ERK/MMP Axis

**Figure 6:**
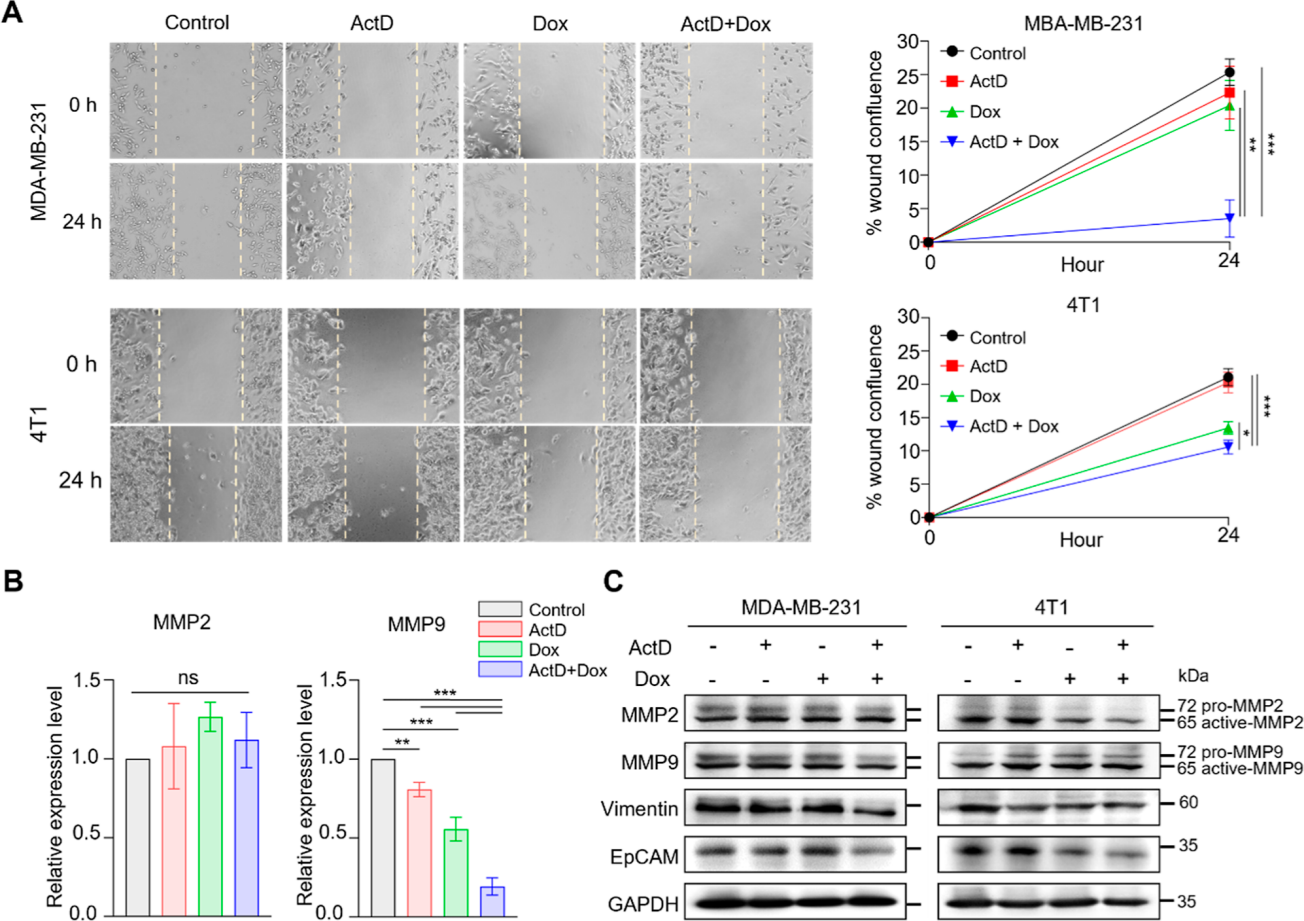
Combination treatment with ActD and Dox inhibits migration and invasion in TNBC cells. (A) In vitro wound healing assay of MDA-MB-231 cells treated with 2 nM ActD and 600 nM Dox and 4T1 cells treated with 0.5 nM ActD and 150 nM Dox for 24 h, *n* = 3 biological replicates. Statistical significance was evaluated using two-tailed *P* values: *P < 0.05, **P < 0.01, and ***P < 0.001 (B) RNA expression levels of MMP2 and MMP9 in MDA-MB-231 cells treated with 2 nM ActD, 600 nM Dox, and 10 ng/mL EGF for 30 h, *n* = 3 biological replicates. *P*-values from one-way ANOVA tests comparing each group are indicated as follows: **P < 0.01, ***P < 0.001, and ns = not significant. (C) MDA-MB-231 and 4T1 cells were treated with ActD (2 nM and 0.5 nM, respectively), Dox (600 nM and 150 nM, respectively), and 10 ng/mL EGF for 36 h. Protein levels of MMP2, MMP9, Vimentin, and EpCAM were detected by Western blotting.

To explore the effect of the combination treatment on TNBC metastasis, we conducted in vitro migration and invasion assays. The results from the wound healing assay demonstrated that the combination of ActD and Dox significantly reduced the migration of MDA-MB-231 and 4T1 cells (Figure 6A). To further assess the effect of migratory and invasive TNBCs, we performed a transwell assay to quantify the number of cells that migrated to the bottom chamber. The combination treatment resulted in a substantial decrease in the migration and invasion capabilities of both MDA-MB-231 and 4T1 cells (Figure S9A,B). The EGFR/ERK/MMP pathway is known to promote tumor progression and metastasis across various cancer types.^37−39^ To evaluate the impact of the combination treatment on these signaling pathways, we analyzed the RNA and protein expression levels following the treatment. The findings revealed that the combination significantly inhibited the expression of MMP9 but not MMP2 in the MDA-MB-231 cells. Moreover, the combination treatment reduced the levels of metastasis-associated markers Vimentin and EpCAM in both TNBC cell lines. Interestingly, in 4T1 cells, the combination suppressed MMP2 expression while having no effect on MMP9 (Figure 6B,C). These results suggest that the combined treatment of ActD and Dox effectively inhibits cell migration and invasion in TNBCs by targeting the EGFR/ERK/MMP signaling axis.

## Discussion and Conclusions

Among the various chemotherapeutic agents, the anthracycline antibiotic Dox is a widely used first-line agent.^40^ It is frequently used in the treatment of breast cancer, bladder cancer, leukemia, etc.^41,42^ The major mechanism of action of Dox is intercalation into DNA, which inhibits the enzyme topoisomerase II, preventing DNA relaxation and leading to DNA damage and cell death.^43,44^ Dox is often used in combination with other chemotherapeutic agents. For example, the combination of Dox with cyclophosphamide is used in the treatment of breast cancer.^45^ However, the effectiveness of Dox is severely compromised by its cardiotoxicity, which leads to acute adverse effects and chronic heart attacks. This necessitates the exploration of new chemotherapeutic options that can maximize the therapeutic benefit of Dox while minimizing recurrence, drug resistance, and toxic effects.

Combining different DNA-targeting agents can increase their target specificity or enhance their sequence recognition ability, thereby potentially improving their anticancer abilities. For instance, the polyamide antibiotic distamycin A has been shown to modulate the DNA alkylation specificity of the potent antitumor antibiotic duocarmycin A with both ligands simultaneously binding to the same DNA target.^46^ We have explored in this study the potential of combining Dox and ActD against TNBC by exploiting their DNA-intercalating properties. Our structural results demonstrate that cooperative binding between these drugs enhances their abilities to intercalate into their respective canonical binding sites. Although cooperative binding has been previously observed in drug–DNA complexes,^47,48^ the ternary structure of the Dox–ActD–DNA complex described here has novel features resulting from the combined effects of these drugs. First, the simultaneous intercalation of ActD and Dox into consecutive DNA sites does not result in steric conflict between the drug molecules since their side chains align perfectly within the minor groove (Figure S10A). In contrast, for the intercalation of individual ActD or Dox molecules into DNA, at least one or two base pairs are required between the preferred intercalation sites to avoid clashes between the two intercalated drug molecules.^14,24^ The orientation of the amino sugar ring of Dox in the current structure differs markedly from that in previously reported individual Dox–DNA complexes. This new binding mode creates an adequate physical space for the insertion of the two different intercalators into consecutive DNA sites without the need for additional flanking base pairs. Additionally, the ternary complex structure demonstrates that ActD and Dox chromophores intercalate into DNA base pairs in a parallel manner, allowing continuous stacking interactions with intercalated base pairs more effectively than in the individual drug complexes (Figure S10B). The staggered arrangement of the two drugs and the opposite orientation of the Dox amino sugar ring promote drug–drug contact and water-mediated drug–DNA interactions, ensuring tighter complex formation with DNA. A comparison of the Dox-binding sites in the current ternary complex structure to individual Dox-DNA complexes underscores the structural effects of the drug combination. While previous Dox complexes exhibit base-pair planarity distortion (average tilt, approximately −11.5° ± 4°), the tilt angle at the Dox-intercalation sites in our ternary complex structure was ca. 1°. Thus, the simultaneous intercalation of ActD and Dox into adjacent DNA sites enhances continuous stacking interactions in drug–DNA complexes, potentially leading to improved therapeutic effects.

ActD has been used to treat cancers such as Ewing’s sarcoma, Wilms’ tumor and rhabdomyosarcoma.^49^ A clinical trial (NCT03669783) is currently underway to evaluate the combination of Dox with other chemotherapeutic agents, including ActD, for the treatment of renal tumors, indicating that the drug’s safety and efficacy had been evaluated. However, the mechanisms of the two DNA-targeting drugs on cancer cells are still unclear. We confirmed the therapeutic effects of the ActD and Dox combination by performing a series of in vitro and in vivo experiments. Our RNA-seq analysis reveals that the combination of ActD and Dox effectively inhibits EGFR expression and protein serine/threonine kinase activity. The observed downregulation of EGFR transcription can be attributed to the synergistic action of both drugs, which target adjacent GC and CG sites within the promoter region. This cooperative interaction disrupts EGFR promoter function, impairing its gene expression. EGFR overexpression in TNBC is associated with aggressive tumor behavior, poor prognosis, and resistance to certain therapies.^50^ In addition, the aberrant activation of the Ras/MAPK pathway is highly prevalent in TNBC.^51,52^ Therefore, inhibiting the EGFR pathway is a validated approach for TNBC treatment. Although several inhibitors targeting the EGFR signaling pathway have been developed,^53^ their use in combination with Dox for TNBC treatment is unknown. In this study, the combination of ActD with Dox exhibited synergistic inhibition of cell growth, migration, and invasion through the EGFR/ERK/MMP pathway in TNBC. In addition, the combination reduces mRNA expression of genes related to tumorigenesis and drug resistance, including c-Myc^54^ and FOXO3a^55^ (Figure S11). In vivo, IHC analysis demonstrated that when Dox is combined with ActD, it significantly decreases EGFR expression and pERK in TNBC tumors in both animal models. In clinical practice, Dox is not normally considered to be targeting EGFR. Previous studies have proposed the combination of Dox with anti-EGFR antibodies against EGFR-expressing tumors to improve the survival of breast cancer patients.^56,57^ This highlights the potential for synergistic therapies targeting EGFR pathways, which could be beneficial for treating EGFR-driven cancers.

Importantly, in our animal studies, we adopted a lower dose (∼20 mg/m^2^) compared to the clinical therapeutic dose of Dox (60–75 mg/m^2^, administered in a 3×-weekly schedule), but we administered it on a weekly schedule. This approach, known as metronomic chemotherapy, has been shown to cause fewer severe adverse effects.^58,59^ Metronomic chemotherapy involves the continuous, low-dose administration of chemotherapeutic agents, which can reduce the toxicity while maintaining therapeutic efficacy. In our study, we found that at lower drug doses in the mouse model, the combination of ActD and Dox consistently demonstrated antitumor activity, prolonged survival rates, and reduced lung and liver metastasis in TNBC mice. Remarkably, this combination outperformed the commonly used AC regimen. Additionally, administering the combination at half the standard dose significantly reduced lymphotoxicity, which is a common side effect of cancer treatment. These results suggest that the combination of ActD and Dox with a metronomic regimen could serve as a cost-effective and safer alternative therapeutic strategy for treating TNBC.

On the other hand, EGFR amplification or mutation is frequently observed in nonsmall cell lung cancer (NSCLC). NSCLC is the most common form of lung cancer, accounting for approximately 85% of all cases. It is a highly heterogeneous disease with distinct molecular subtypes, and its management has evolved significantly with the advent of targeted therapies. One of the most significant advancements in the treatment of NSCLC has been the use of tyrosine kinase inhibitors (TKIs), particularly in patients with mutations in the EGFR. TKIs such as erlotinib, gefitinib, afatinib, and osimertinib have been widely used and have shown significant efficacy in patients with EGFR-sensitive mutations. Despite their initial success, drug resistance remains a major challenge in the control of NSCLC. Thus, we propose combining ActD, Dox, and TKI treatments in NSCLC, which can simultaneously inhibit EGFR transcription and target the tyrosine kinase domain of proteins. Moreover, combination therapy may reduce the occurrence of drug resistance. Future applications will require further experimental validation.

In summary, we have successfully demonstrated the structural basis and the target validation of combining two DNA-intercalators, ActD and Dox. Our structural analyses emphasize the importance of these two intercalating drugs for simultaneous targeting to DNA with increased sequence specificity and provide a mechanistic basis for combination chemotherapy with DNA-targeting drugs.

## Experimental Section

### Chemicals and Oligonucleotides

Actinomycin D (catalog no. SI-A1410), doxorubicin (catalog no. SI-D1515), cisplatin (catalog no. SI-P4394), 5-fluorouracil (catalogue no. SI-P6627), and cyclophosphamide (catalog no. EP-C3250000) were purchased from Sigma-Aldrich (St. Louis, MO). All drugs were dissolved in appropriate solvents according to the manufacturer’s instructions to prepare working stocks. All other chemicals used in this study were of molecular grade with >95% purity. DNA oligonucleotides for biophysical and crystallization studies were commercially synthesized by MDBio Ltd., Taiwan, and purified using polyacrylamide gel electrophoresis. Oligonucleotide stocks were prepared by dissolving lyophilized samples in double-distilled H_2_O, and their concentrations were determined by using a JASCO-v630 UV–visible spectrophotometer (JASCO International, Tokyo, Japan). Beers’ law was used to calculate the oligonucleotide concentration as follows: *A* = ϵ*bc* (*A*, absorbance at 260 nm; ϵ, molar extinction coefficient; *b*, cell path length; and *c*, molar concentration).^60^ The approximate value of ϵ260 nm for each oligonucleotide was determined using https://molbiotools.com/dnacalculator.html.

### X-ray Crystallography

To obtain ActD–Dox–DNA complex crystals, 0.125 mM d(AGCCGT)_2_ oligonucleotide duplex (5′-AGCCGT-3′/5′-ACGGCT-3′) was cocrystallized with ActD and Dox in a 1:1:1 ratio using a sitting-drop vapor-diffusion method at 20 °C. Long, needle-shaped, orange crystals grew within 3 days in a 5 μL crystallization drop containing 30 mM LiCl, 3 mM MnCl_2_, 25 mM MES (pH 6.5), and 3% PEG400 at 20 °C. A single crystal was used to collect X-ray diffraction data at the synchrotron facility of the National Synchrotron Radiation Research Center (Taiwan) at the BL15A1 beamline. Integration, reduction, and scaling of the crystallographic data were performed using the HKL2000 software package.^61^ Phase determination was performed by molecular replacement (MR) of phaser MR in a Python-based hierarchical environment for integrated Xtallography (PHENIX, v1.10.1), and refinements were performed using Phenix.refine in the PHENIX suite.^62^ To determine the phases for the ternary complex, partial structures of Dox-d(CGATCG)_2_ complex (PDB ID: 1D12) and ActD-Echi-d(ACGTGCT)_2_ complex (PDB ID: 7DQ0) were used as the initial templates. The crystallographic object-oriented toolkit (COOT) v0.8.9.2 was used for model generation and the addition of water molecules based on electron-density maps.^63^ The final crystallographic and refinement statistics of the complexes are listed in Table S6. DNA base-pair and base-pair step parameters were analyzed using the online server Web3DNA.^64^ The DNA torsion angles, local base pairs, and base-pair step parameters are given in Table S7.

### CD Spectroscopy

The oligonucleotide sequences used for CD spectroscopy are listed in Supporting Information Table S3. DNA duplexes (20 μM) were prepared in a buffer containing 50 mM sodium cacodylate (pH 7.2) and 5 mM MgCl_2_. Oligonucleotide samples were heated at 95 °C for 5 min and then annealed at 4 °C for 30 min. Later, different ratios of ActD and Dox alone and their combinations were added to the oligonucleotide samples and incubated for 24 h. CD spectra of DNA oligonucleotides were recorded between 320 and 220 nm at 1 nm intervals by using a Chirascan V100 circular dichroism spectrometer, as described earlier.^65,66^

### DNA Thermal Stability Measurement

The stability of DNA was measured by determining the melting temperature (*T*_m_) of DNA in the presence and absence of drugs and by acquiring the UV absorbance versus temperature profile.^47^ Oligonucleotide duplexes (3 μM) were prepared in a buffer containing 50 mM sodium cacodylate (pH 7.2) and 5 mM MgCl_2_. To prepare the samples, d(AAAAGCCGAAAA)_2_ oligonucleotides were denatured at 95 °C for 5 min and annealed on ice for 30 min. Later, ActD (3 μM) or Dox (3 μM) alone or in combination (3 μM each) were added, and the samples were incubated for 24 h at 4 °C. Melting temperature profiles were recorded at 260 nm by gradually raising the temperature from 4 to 95 °C. *T*_m_ values were determined by polynomial fitting to the curves using Varian\Cary WinUV Thermal v3.00 (Agilent, Santa Clara, CA, USA). Changes in the melting temperature (Δ*T*_m_) were calculated as the difference between the *T*_m_ values of the DNA duplex in the presence and absence of the drug.

### Fluorescence Spectroscopy and Kinetic Analysis

d(AAAAGCCGAAAA)_2_ oligonucleotides were prepared in a 50 μM Na cacodylate buffer (pH 7.3) with 5 μM MgCl_2_ in ddH_2_O, heated at 95 °C for 5 min, and then cooled at 4 °C for 30 min. For fluorescence experiments, 1 μM of DOX and ActD solutions (in ddH_2_O) were added to various concentrations of dsDNA solution to a final volume of 300 μL. Fluorescence measurements were performed using the Jasco FP-8300 Spectrofluorometer with an excitation wavelength of 481.0 nm and a measurement range of 510–700 nm. The Hill slope and *K*_d_ value were determined from the fluorescence intensity at 560 nm. For the calculation of the Hill slope and the *K*_d_, fluorescence binding data were fitted using the Hill equation: *Y* = *B*_max_ × *X*^*h*^/(*K*_d_^*h*^ + *X*^*h*^), where *Y* represents the fluorescence quenching ratio, *B*_max_ is the maximum binding capacity, *X* is the quencher concentration, *K*_d_ is the dissociation constant, and *h* is the Hill coefficient. Nonlinear regression was performed by using GraphPad Prism 8.0.2 to determine the binding parameters.

### Cell Lines and Proliferation Assay

The MDA-MB-231 and MCF-7 cell lines were purchased from the Bioresource Collection and Research Center (BCRC, Taiwan). The 4T1 cell line was provided by Dr. Chih-Rong Shyr (China Medical University, Taiwan). Cells were incubated in DMEM (MDA-MB-231) or RPMI1640 (MCF-7 and 4T1) supplemented with 10% FBS and 1% antibiotic-antimycotic in a humidified atmosphere with 5% CO_2_ at 37 °C. Dose-dependent inhibition of proliferation was determined by the MTT assay to calculate IC_50_ values using a nonlinear curve-fitting analysis using GraphPad Prism v8.0.0.

### SynergyFinder Analysis and CI Assay

Synergy between the two drugs was determined by SynergyFinder (https://synergyfinder.fimm.fi) and CI assays.^67,68^ For the SynergyFinder analysis, different concentrations of ActD (1 to 10 nM) and Dox (0.1 to 1 μM), and their combination in varying ratios were applied according to IC_50_ values. The synergy scores were quantified using the zero interaction potency (ZIP) model, where the expected effect is calculated as *E*_expected_ (*x*,*y*) = *E*_1_ (*x*) × *E*_2_ (*y*). The CI was determined using CompuSyn 1.0 with different combination ratios of ActD and Dox (1:100 to 1:500), with CI calculated as CI = *D*_1_/*D*_1_^(*m*)^ + *D*_2_/*D*_2_^(*m*)^, where *D*_1_ and *D*_2_ are the doses of ActD and Dox that achieve the same effect when used alone, and *D*_1_^(*m*)^ and *D*_2_^(*m*)^ are the doses required in combination to achieve that effect. CI = 1, CI < 1, and CI > 1 represent additive, synergistic, and antagonistic effects, respectively.^69^

### Cell Cycle Analysis

MDA-MB-231 and 4T1 cells (2 × 10^5^ cells/well) were treated with ActD (0.25 nM) and Dox (75 nM) in a 1:300 ratio in 6-well plates for 24 h. After 24 h of incubation, the cells were resuspended by trypsin and then fixed with 75% ethanol at 4 °C. Samples were stained with 200 μL of propidium iodide (PI) buffer containing 0.03% Tween 20, 0.05 mg/mL PI (P1304MP), and 0.1 mg/mL RNase (EN0531) and allowed to react in the dark for 15 min. Cell cycle distribution was quantified using a flow cytometer (BD Biosciences), and data processing and image generation were performed using FlowJo V10.

### RT-qPR Analysis

Cells were treated with the indicated dosage of individual ActD, Dox, or a combination of both. Total RNA was extracted by using the Trizol reagent (Invitrogen, 15596026) and dissolved in DEPC water to generate cDNAs using the PrimeScript RT Reagent Kit (TaKaRa, RR037A). The cycling procedure was 95 °C for 10 min, 95 °C for 10 s, and 60 °C for 30 s for 40 cycles. Target gene expression was normalized to that of GAPDH. The primer sequences used for this experiment are listed in Table S8.

### RNA-Sequencing Analysis

RNA was extracted as described above and stored at −80 °C for further RNA-seq analysis. The quality of the RNA samples was determined by using a Qsep 100 DNA/RNA Analyzer (BioTools Co., Ltd.). Before sequencing the raw data, quality control was performed, and the RNA quality number (RQN) ≥ 7.6 was set for further analysis. Paired-end sequencing with 150 bp reads was performed on an Illumina NovaSeq 6000 instrument to sequence libraries.^70^ Two biological replicates were used for each group. The resulting data, which consisted of high-quality clean reads, were used for further analyses. The read pairs from each sample were aligned to the reference genome (e.g., *H. sapiens*, GRCh38) using the HISAT2 software tool (http://daehwankimlab.github.io/hisat2/). The read counts mapped to each gene were determined using feature counts.^71^ For gene expression analysis, the “trimmed mean of M-value” normalization was performed using DEGseq2 without biological duplicates. In addition, “Relative Log Expression” normalization was performed with DESeq2 with biological duplicates. DEGs between the two conditions were identified using DEGseq (without biological replicates) and DESeq2 (with biological replicates). DEGseq uses a negative binomial distribution model, whereas DESeq2 uses a Poisson distribution model. The resulting *P*-values were adjusted using Benjamini and Hochberg’s approach to control the false discovery rate. To investigate the functional enrichment of DEGs, GO, and KEGG pathway analyses were performed by using the ClusterProfiler package. Disease Ontology (DO) terms were mapped to MeSH, ICD, NCI thesaurus, SNOMED, and OMIM using the DOSE package. Gene Set Enrichment Analysis (GSEA) was performed with 1000 permutations using the Molecular Signatures Database (MSigDB) to identify enriched biological functions and activated pathways.

### MDA-MB-231 Xenograft Model

Animal experiments were conducted according to the guidelines approved by the Institutional Animal Care and Use Committee (IACUC nos. 109-024R and 109-047) of National Chung Hsing University. MDA-MB-231 cells (5 × 10^6^ cells suspended in 100 μL of culture medium and Matrigel in a 1:1 ratio) were injected subcutaneously into the right flank of female 10–12 week-old BALB/C/Athymic NCr-nu/nu mice (National Laboratory Animal Center, Taiwan). Once the tumor volume reached >50 mm^3^, the mice were divided into different groups (*n* = 6 per group): Control (PBS), ActD (20 μg/kg), Dox (0.5 mg/kg), a combination of ActD (20 μg/kg) and Dox (0.5 mg/kg), or a half-dose combination of ActD (10 μg/kg) and Dox (0.25 mg/kg) administered once a week for eight consecutive weeks. The weight and tumor size of the mice were measured twice weekly. Tumor dimensions were measured using a digital caliper, and tumor volume (mm^3^) was calculated using the following formula: length × (width)^2^ × 0.5. The mice were euthanized with carbon dioxide 56 days after treatment, and the tumors and livers were removed for further analysis.

### 4T1 Orthotopic Breast Cancer Model

For the orthotopic breast cancer model, 5 × 10^5^ 4T1 cells suspended in 100 μL of PBS were injected into the fourth right mammary fat pad of female 6–8 week-old immunocompetent BALB/c mice. Treatment was initiated when the tumor volume reached >30 mm^3^. The mice were divided into seven groups: Control (PBS), ActD (20 μg/kg), Dox (0.5 mg/kg), a combination of ActD (20 μg/kg) and Dox (0.5 mg/kg), a half dose in combination of ActD (10 μg/kg) and Dox (0.25 mg/kg), cyclophosphamide (CP) (5 mg/kg), or a combination of CP (5 mg/kg) and Dox (0.5 mg/kg) once a week for four consecutive weeks. After 28 days, the mice were sacrificed and euthanized, and their tumors and organs were removed for further analysis. The survival ratio of 4T1 tumor-bearing mice was evaluated in accordance with the IACUC recommendations, and mice were euthanized when the tumor volume exceeded 1000 mm^3^ or they showed signs of dyspnea resulting from cancer metastasis to the lungs.

### Hematology Analysis

Blood was collected from the mice by cardiac puncture after euthanization and stored in ethylenediaminetetraacetic acid-containing blood collection tubes. CBC was performed by using a hematology analyzer. The number and percentage of various white blood cells, including neutrophils, lymphocytes, monocytes, basophils, and eosinophils, were recorded.

### Hematoxylin and Eosin and Immunohistochemistry Staining

Mouse tumor and lung biopsy specimens were fixed in 10% neutral-buffered formalin. Paraffin-embedded tissue sections were used for hematoxylin, eosin, and IHC staining. For IHC staining, 1:250 EGFR monoclonal antibody (Zeta Corporation #ZR16), 1:200 *K*_i_-67 recombinant monoclonal antibody (Thermo #MA5-14520), and 1:200 pERK (Thr202/Tyr204) antibody (Cell Signaling Technology #9101) were used. The percentage of Ki67-positive cells was estimated in four tumor regions per mouse at ×400. To evaluate EGFR and pERK expression, the *H*-scores for EGFR- and pERK-expressing tumor cells were calculated based on the staining intensity. Staining intensity values were 0 (no evidence of staining), 1 (weak), 2 (moderate), and 3 (strong). *H*-score was calculated as follows: *H*-score = (0 × *P*0) + (1 × *P*1) + (2 × *P*2) + (3 × *P*3), where *P* represents the percentage of stained cells.

### Western Blot Assay

The concentration of cell lysis proteins was quantified using the Bradford assay (Bioshop). Protein samples (25 μg) were loaded onto a 10% SDS-PAGE gel and transferred onto a PVDF membrane. The membranes were blocked with 5% BSA (Sigma-Aldrich) at room temperature for 1 h. Primary antibodies were diluted in TBST buffer (Table S9) and incubated at 4 °C overnight. The next day, the membranes were incubated with HRP-conjugated secondary antibodies at room temperature for 1 h. Chemiluminescence signals were detected using the UVP ChemStudio imaging system with an ECL reagent (BIO-HELIX). For reprobing the membranes, stripping buffer (StripPRO, SP01-500) was used to remove the antibodies, followed by blocking, adding primary and secondary antibodies, and imaging, as described above.

### Luciferase Reporter Assay

The promoter region sequences from −604 to −10 bp upstream to the EGFR transcription start site for both wild-type and mutant EGFR were synthesized by GeneDirex, Inc. (Taipei, Taiwan). These promoter sequences were cloned into the LVR-1048-pLV-promoterless-firefly luciferase-PGK-Puro vector (Cellomics Technology, LLC) and sequenced for verification. Cells were transfected with the plasmid using a lentiviral expression system. Detecting firefly luciferase activity was according to the manufacturer’s instructions (Thermo, 16176). Cells were seeded at a density of 2 × 10^4^ cells per well in a 96-well plate and incubated overnight at 37 °C in 5% CO_2_. After the indicated treatment for 16 h, the culture media was aspirated from the cells, and the cells were rinsed once with DPBS. A volume of 50 μL per well of cell lysis buffer was added to each well. A volume of 20 μL of cell lysate from each well was transferred to a 96-well plate. 50 μL of the firefly luciferase working solution was added to each well, and the luminescence was then measured using a luminometer (Soft Max Pro v5.4.6).

### Wound Healing Assay

1 × 10^4^ 4T1 and MDA-MB-231 cells were seeded in inserts in 24-well plates. After 18 h, the insets were removed, and images were taken as the initial time point. The cells were then treated with ActD and Dox for 24 h, and then pictures were taken. The percentage of wound confluence was calculated using the formula: ((*A* – *B*) × 100%)/*A*, where *A* is the width of the initial wound and *B* is the distance of the wound after 24 h.

### Transwell Migration and Invasion Assay

4T1 and MDA-MB-231 cells were seeded at a density of 1 × 10^5^ cells per well in the upper chambers of transwell inserts with 200 μL of serum-free medium. For the invasion assay, the transwell filters were coated with 50 μL of matrigel diluted at a 1:5 ratio with serum-free medium. The lower chambers were loaded with 500 μL of medium containing 10% FBS and the indicated concentration of the compound. After 24 h, the upper chambers were removed and further incubated for 16 h until the cells attached to the bottom of the well. Then, the cells were fixed with 4% PFA and stained using Giemsa staining. To assess cell migration, three randomly chosen low-magnification fields (40×) were photographed under a microscope.

### Statistical Analysis

Differences among groups were compared using two-tailed Student’s *t* tests to calculate *P*-values. The qPCR data were evaluated using one-way analysis of variance. Statistical significance was indicated as follows: *P < 0.05; **P < 0.01; ***P < 0.001, with “ns” denoting nonsignificance. The data presented in the bar graphs show the mean ± standard deviation or mean ± standard error of the mean. All statistical graphs were generated using GraphPad Prism v5.01 (San Diego, CA, USA).

## Supporting Information Available

The newly determined crystal structure has been assigned to the ActD-Dox-DNA complex (PDB ID: 9JL7). The authors will release the atomic coordinates upon article publication. RNA-seq data, in vivo study, IHC stain, and X-ray crystallographic and refinement statistics of structure (PDF)
Molecular formula strings (CSV)

## Abbreviations

ActD: actinomycin D
Dox: doxorubicin
EGFR: epidermal growth factor receptor
