# Supplementary material for: Structural and Functional Insights into Targeting GCCG Sites in the EGFR Promoter by Two DNA Intercalators to Inhibit Breast Cancer Metastasis
Source: J Med Chem. 2025 Mar 3;68(6):6601–15. doi: 10.1021/acs.jmedchem.4c03203 (PMC11956004; doi:10.1021/acs.jmedchem.4c03203)
Supplement: Supplementary file 1 — jm4c03203_si_001.pdf [file jm4c03203_si_001.pdf]

Supporting Information

Structural and functional insights into targeting GCCG sites in the EGFR promoter by two DNA intercalators to inhibit breast cancer metastasis

Chih-Chun Chang[1,†], Hsin-Ju Li[2,†], Roshan Satange[2,†], Shan-Meng Lin[2], Tai-Lin Chen[3], Chi-Chien Lin[4],Stephen Neidle[5], and Ming-Hon Hou[1,2,6,*]

[1] Graduate Institute of Biotechnology, National Chung Hsing University, Taichung, 402, Taiwan.
[2] Graduate Institute of Genomics and Bioinformatics, National Chung Hsing University, Taichung, 402, Taiwan.
[3] Post Baccalaureate Medicine, School of Medicine, National Chung Hsing University, Taichung, 402, Taiwan.
[4] Institute of Biomedical Science, National Chung Hsing University, Taichung, 402, Taiwan.
[5] The School of Pharmacy, University College London, London, WC1N 1AX, United Kingdom.
[6] Biotechnology Center, National Chung Hsing University, Taichung 402, Taiwan.

* To whom correspondence should be addressed. Tel: +886 4 2284 0338 (Ext 7011); Fax: +886 4 2285 9329 ; Email: mhho@nchu.edu.tw

† Joint First Authors.

Table of contents

Figure S1. Biophysical measurements of ActD and Dox in GCCG-containing DNA S2

Figure S2-3. Crystal structure DNA parameters in the ActD-Dox-d(AGCCGT)$_2$ complex S3-4

Figure S4. Cell viability and Cell cycle analysis for Dox and ActD S5

Figure S5. Protein-protein interaction (PPI) network analysis from RNA-seq results S6

Figure S6. Gene ontology analysis for Dox and ActD S7

Figure S7. Sequences of EGFR (ENSG00000146648) promoter region S8

Figure S8. In vivo study for Dox and ActD S9

Figure S9. Migration and invasion assay for Dox and ActD S10

Figure S10. Comparison of drug-drug interactions and direction of intercalated chromophores in DNA-ligand complexes. S11

Figure S11. RT-qPCR analysis for Dox and ActD S12

Table S1. List of sequences used for biophysical analysis. S13

Table S2-4. In vitro cell viability assay S13-16

Table S5. Hematological analysis for Dox and ActD S17

Table S6-7. Crystallographic and refinement statistics of ActD-Dox-d(AGCCGT)$_2$ complex S18-19

Table S8-9. Experimental materials for RT-qPCR and Western blot assays S20-21

Supplementary Figures

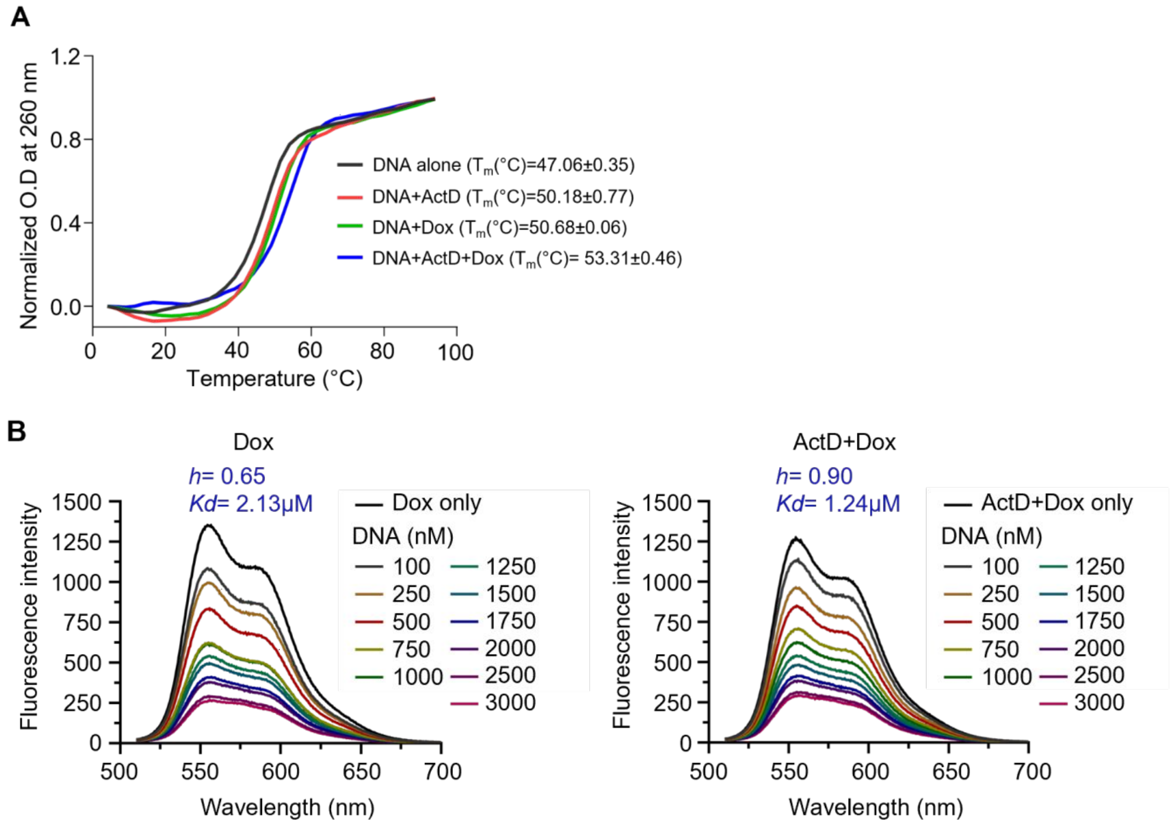

Figure S1. The combination of Dox and ActD increased the Tm and of GCCG-containing DNA. (A) T_m curves shows the combination of ActD and Dox enhances the stability of -GCCG-motif containing DNA sequence. **(B)** Fluorescence emission spectra of DOX and ActD–DOX in the presence of GCCG-containing dsDNA at various concentrations. The initial concentration of both DOX and ActD was 1 µM. The Hill slope (*h*) and *Kd* value was determined from the fluores-cence intensity at 560 nm, calculated based on specific binding with the Hill slope.

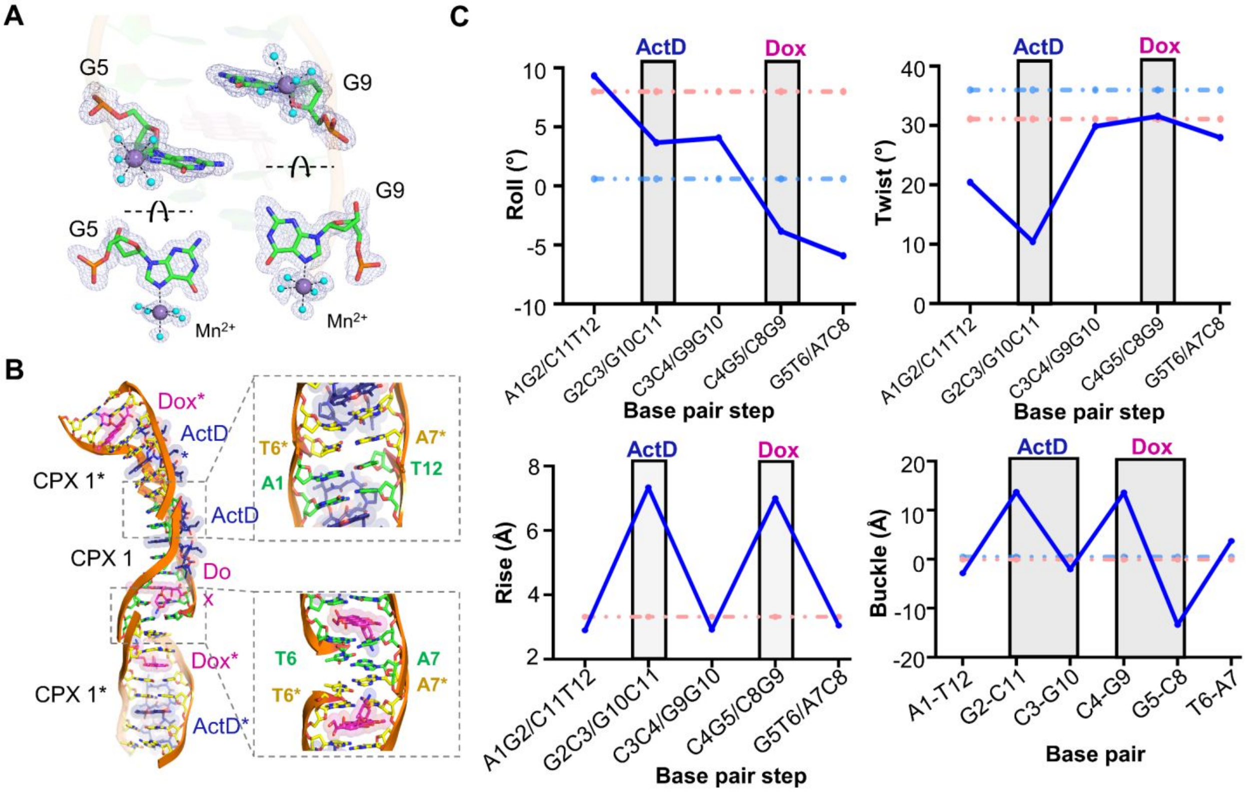

Figure S2. Structural changes in DNA upon ActD and Dox intercalation. (A) Metal ion coordination details observed in the current crystal structure. Octahedral coordination of two manganese (Mn^{2+}) ions with guanine bases on two strands near Dox binding is shown. One Mn^{2+} shows octahedral coordination with G5, while the other Mn^{2+} stabilizes the G9 base. Mn^{2+} ions are represented in blue and waters are shown in cyan spheres. The $2|F_0| - |Fc|$ electron density maps (contoured at 1.0 σ) are shown in gray mesh. **(B)** Details of crystal packing interactions in the current structure exhibit an end-to-end duplex-duplex stacking interaction. Symmetry-related residues are indicated by asterisk. **(C)** DNA parameters in the current complex structure of ActD-Dox-d$(AGCCGT)_2$. Changes in DNA roll (º) and twist (º) parameters at different base pair steps and changes in the rise (Å) and buckle (º) parameters are shown.

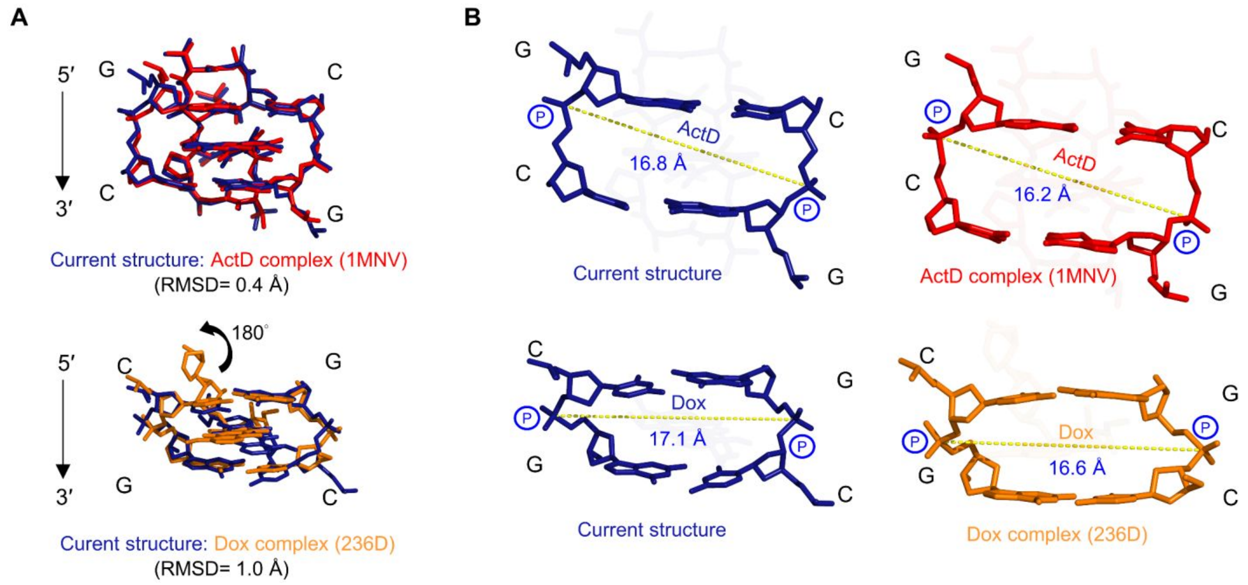

Figure S3. Binding site comparison and changes in interstrand phosphate-phosphate (P-P) distances in the previous and current complexes. (A) Superimposition of the ActD and Dox binding sites from the current complex structure (blue sticks) with the previously reported ActD-d(ATGCTGCAT)$_2$ complex (red sticks) and Dox-d(CGTACG)$_2$ (orange sticks) showing root mean square deviations (RMSD) of 0.4 and 1.0 Å, respectively. **(B)** The increase in interstrand P-P distances at ActD and Dox binding sites in the current structure (blue sticks) compared to previous ActD-DNA (red sticks) and Dox-DNA (orange sticks) complex structures.

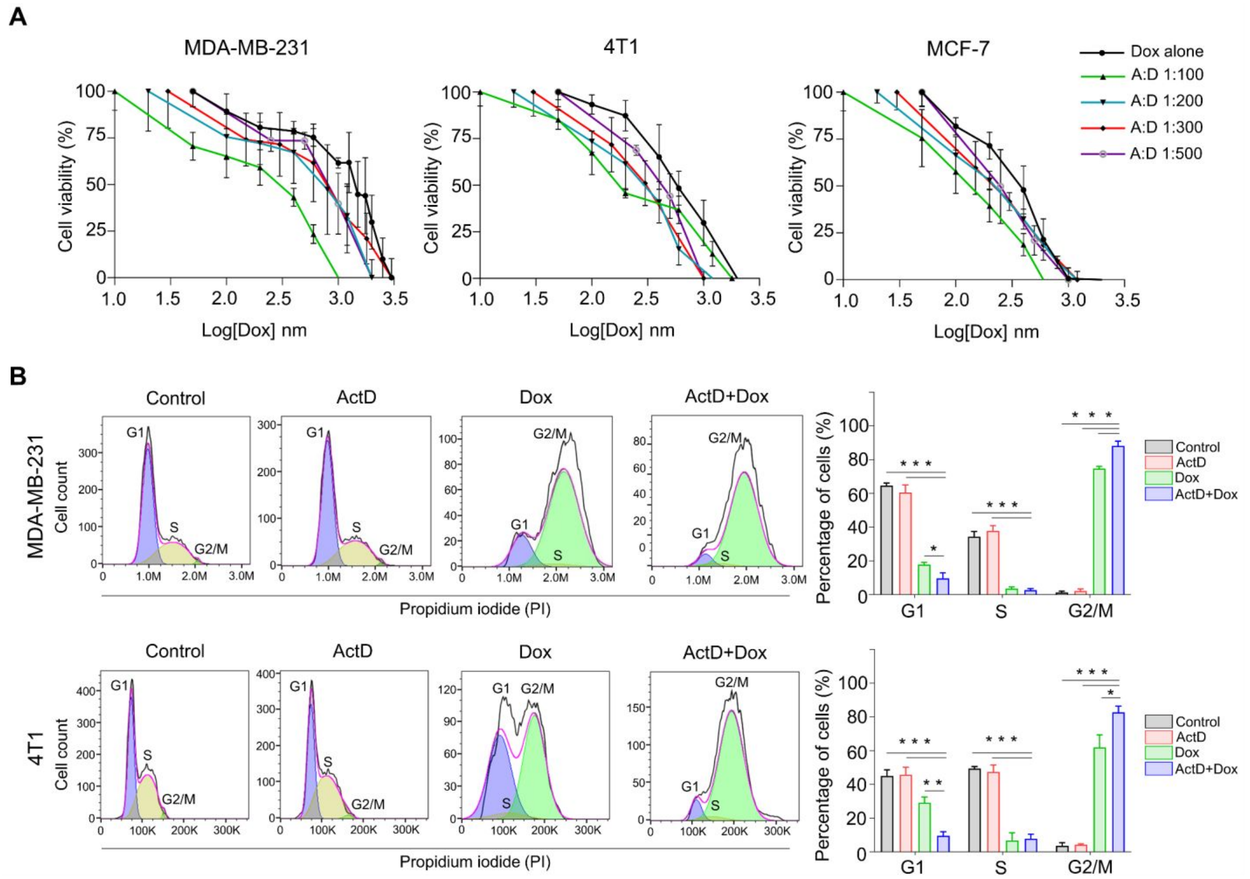

Figure S4. Synergistic effects of Dox and ActD combination against breast cancer cells. (A) Dose–response curves for the different ratios of ActD and Dox treatments on breast cancer cells (n=3 biological replicates). The data is expressed as the mean ± SD (n=3). **(B)** *In vitro* combination of ActD and Dox exhibits a synergistic effect on TNBCs. Cell cycle analysis of TNBCs treated with 1:300 ratio molar concentration of ActD and Dox for 24 h and then stained with propidium iodide. The DNA content was analyzed by flow cytometry and quantified the cell cycle distribution by FlowJo V10 (n=3 biological replicates).

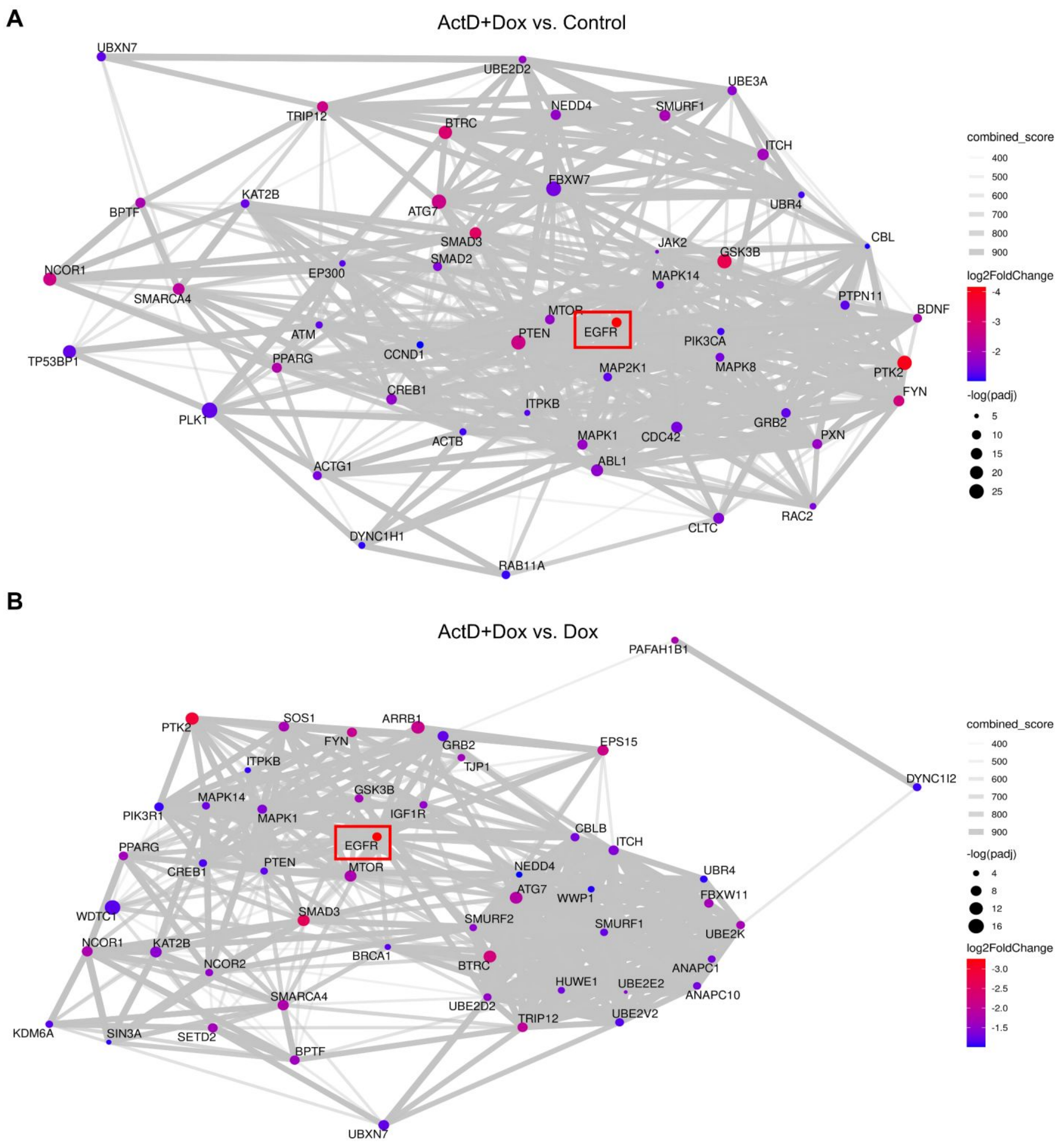

Figure S5. Protein-protein interaction (PPI) network analysis from RNA-seq results. (A) PPI analysis comparing the combination group with the control and **(B)** Dox groups.

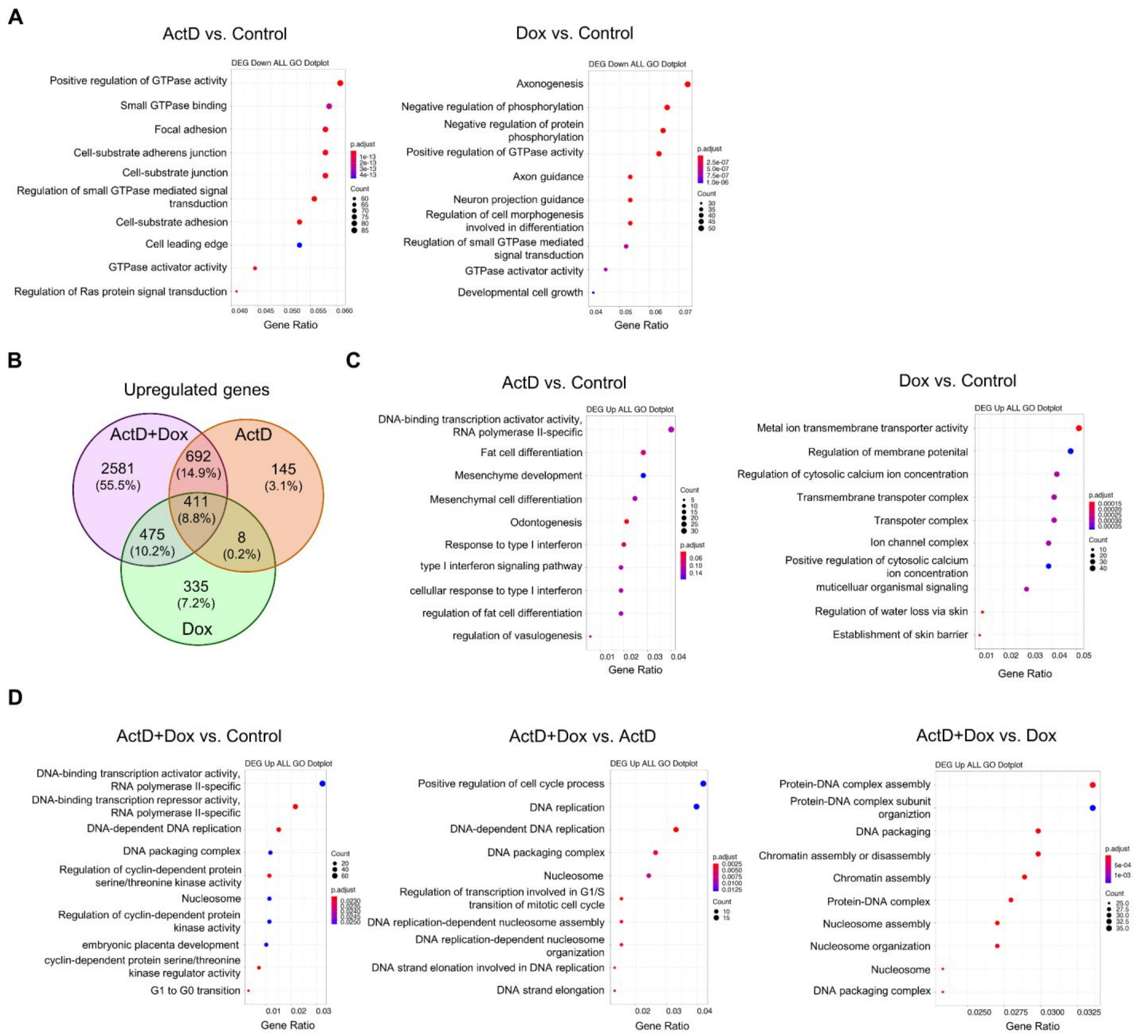

Figure S6. Gene ontology (GO) analysis of differentially expressed genes upon ActD, Dox and the combination treatment. **(A)** GO analysis of downregulated genes after ActD and Dox treatment for 24 h. **(B)** Venn diagram displaying the overlap of statistically significant upregulated differentially expressed genes. **(C)** GO analysis of upregulated genes ActD and Dox treatment and **(d)** the comparison of the combination treatment with single drug treatment. The GO terms encompassed three biological domains: cellular component, biological process, and molecular function.

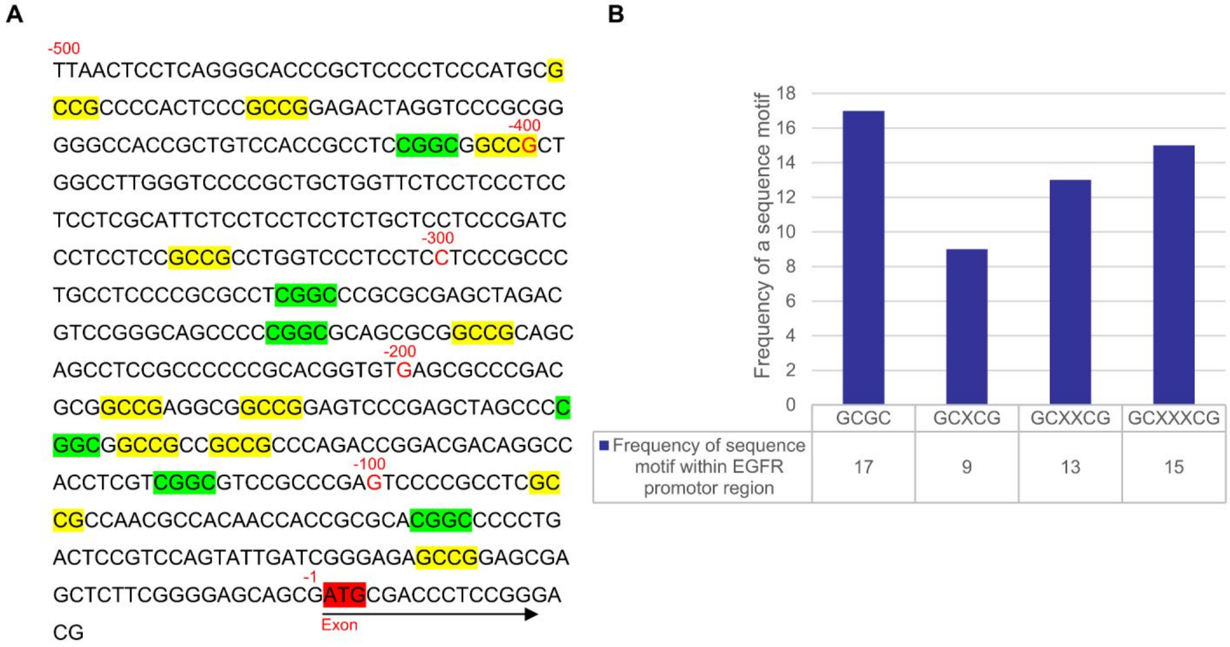

Figure S7. Sequences of EGFR (ENSG00000146648) promoter region. (A) Representation of the EGFR promoter region from -500 to -1. The arrow to the right indicates the start of exon 1. The 5′-GCCG-3′ motifs containing zero (0) flanking bases between the preferred binding sites of ActD and Dox (5′-GC and 5′-CG, respectively) are highlighted in yellow (for the sense strand) and green (for the antisense strand). **(B)** The frequency of occurrence of GCCG (with 0 bases between the preferred intercalation sites of ActD and Dox), GCXCG (with at least 1 base between the preferred intercalation sites of ActD and Dox), GCXXCG (with at least 2 bases between the preferred intercalation sites of ActD and Dox), GCXXXCG (with at least 3 bases between the preferred intercalation sites of ActD and Dox), wherein X stands for one of the four bases A, T, G or C, is shown. The GC-rich promoter region of EFGR contains a substantial number of continuous GCCG sites that are ideal for the simultaneous binding of ActD and Dox to DNA.

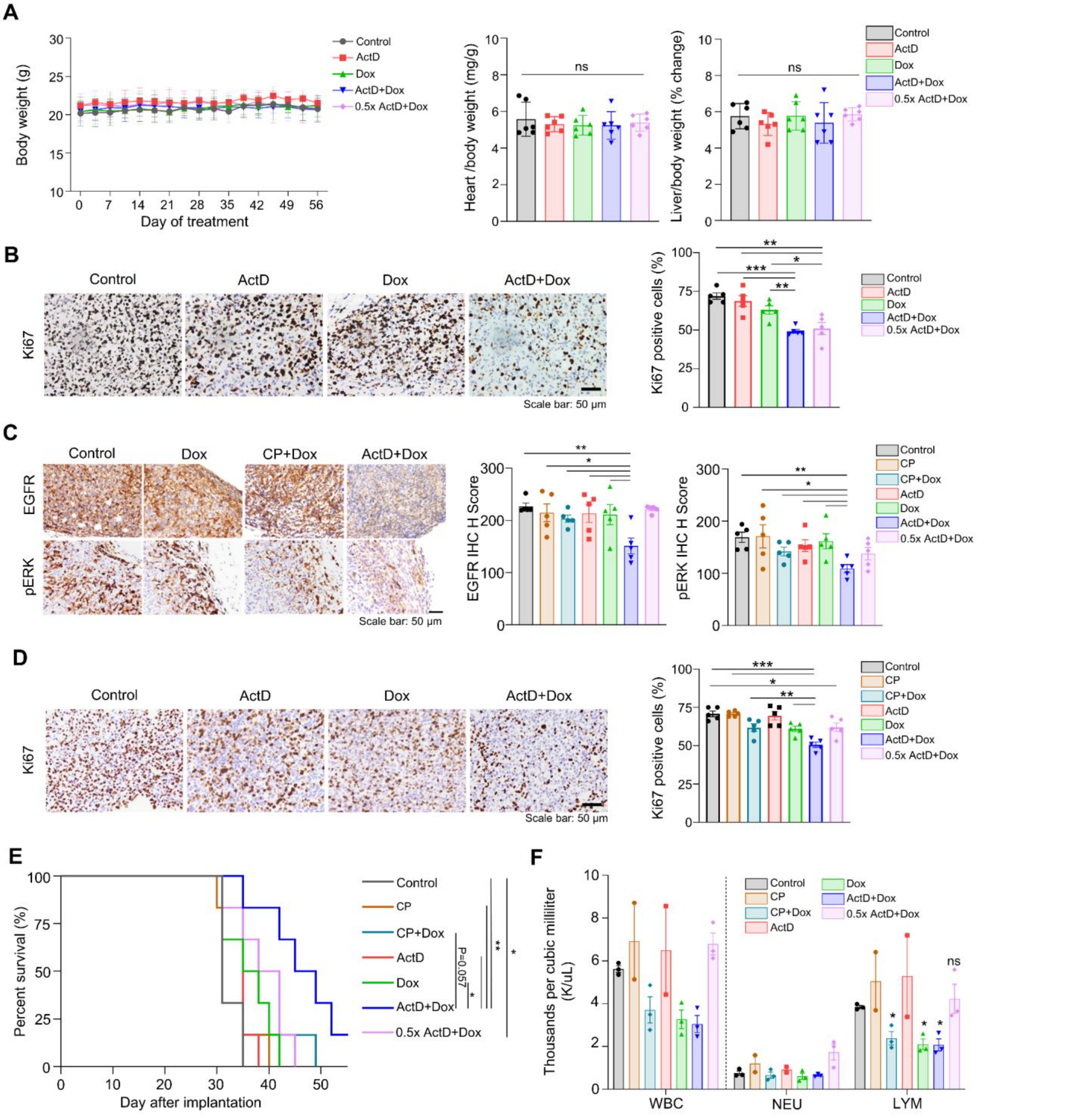

Figure S8. Combination of ActD and Dox suppresses tumor growth in MDA-MB-231 xenograft and 4T1 orthotropic TNBC model. (A) Changes in body weight, heart and liver weight of mice after each treatment are presented as mean ± SD. 'ns' indicates no significant difference. **(B)** Immunohistochemistry staining and statistics results of Ki67 expression in MDA-MB-231. **(C)** IHC staining and statistical results of EGFR and pERK on 4T1 tumor sections (n = 5 per group), Scale bar: 50 μm. Data is presented as mean ± SEM (*P<0.05, **P<0.01, ***P<0.001). **(D)** Immunohistochemistry staining and statistics results of Ki67 expression in 4T1 tumor sections (n = 5 per group). The data are presented as mean ± SEM (*P<0.05, **P<0.01, ***P<0.001). Scale bar: 50μm. **(E)** Survival curve for mice bearing 4T1 model (n = 6 per group). The survival analyses were statistically significant, as determined by log-rank test (*P<0.05, **P<0.01). **(F)** The number of white blood cells (WBCs) in BALB/c mice was analyzed using a complete blood count. Data are presented as mean ± SEM (*P<0.05). Abbreviations: WBC, white blood cells; NEU, Neutrophils; LYM, Lymphocytes.

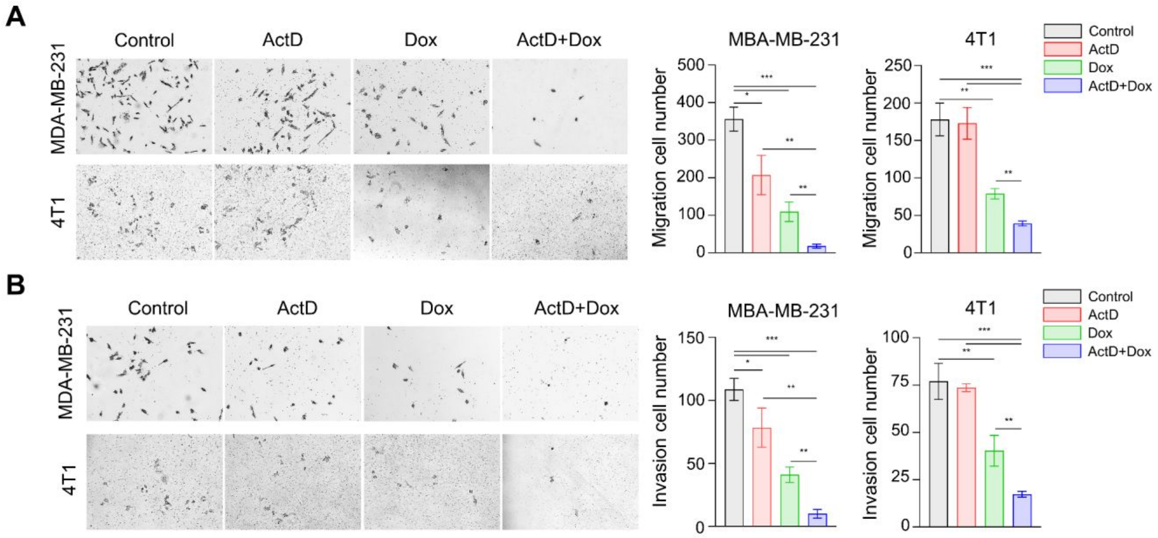

Figure S9. Combination treatment with ActD and Dox inhibits migration and invasion in TNBC cells. (A) Cell migration and **(B)** invasion activities of MDA-MB-231 and 4T1 cells were assessed using a transwell assay under the same drug concentrations and treatment durations as wound healing assay. Images were captured at 40x magnification. All data are presented as mean ± SD, n = 3 biological replicates, and statistical significance is determined using a two-tailed test: *P < 0.05, **P < 0.01, ***P < 0.001.

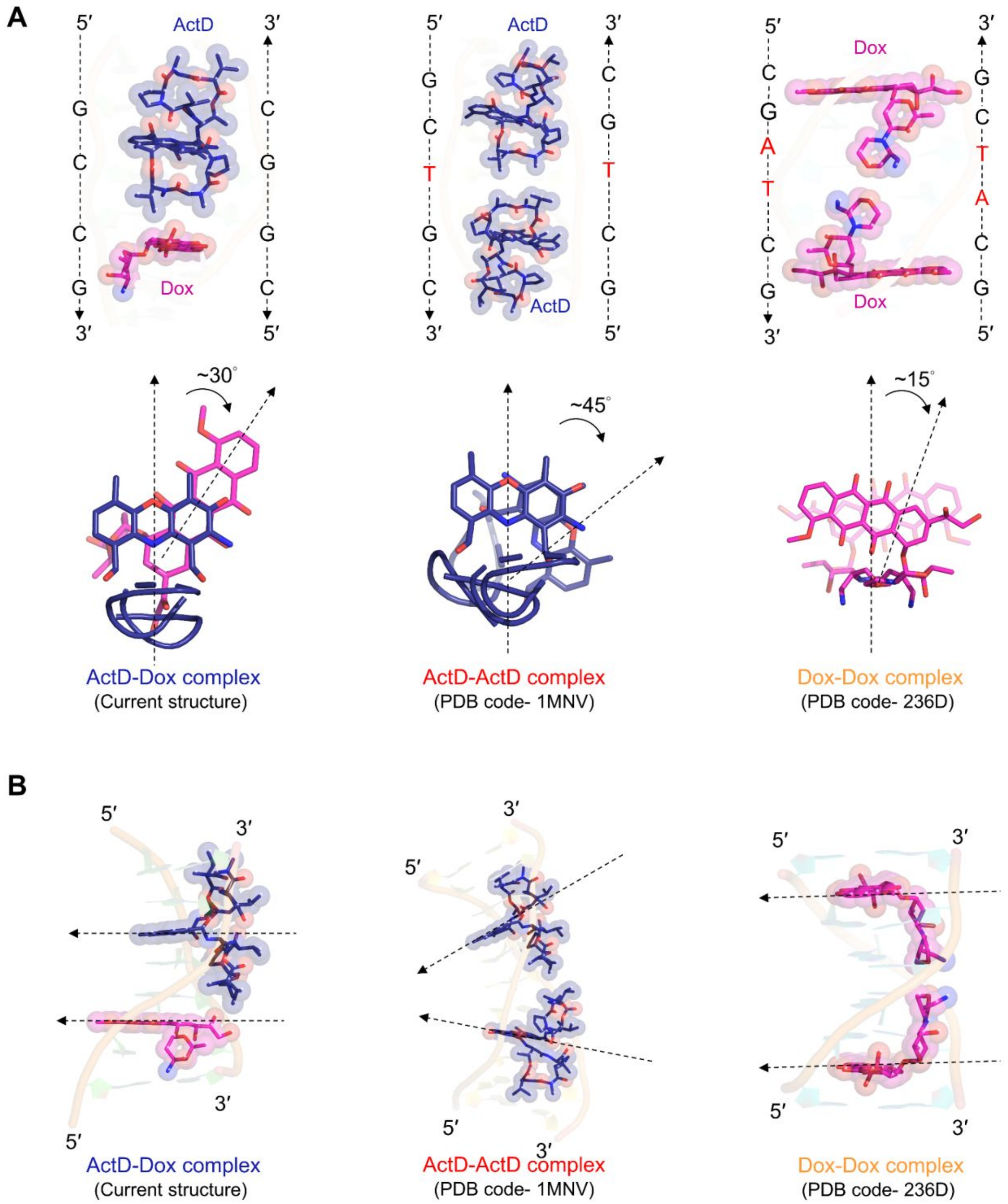

Figure S10. Comparison of drug-drug interactions and direction of intercalated chromophores in DNA-ligand complexes. (A) Continuous GCCG motif does not cause steric clashes between two different drugs, actinomycin D (ActD) and doxorubicin (Dox) upon simultaneous intercalation as observed in the current complex structure. For intercalation of two ActD or two Dox in a same DNA, at least one or two base pairs must present between the preferred binding sites of the drugs to show cooperative binding and avoid steric clashes between drugs. The different staggering angles formed by two drug molecules in these structures of DNA-ligand complex are shown. **(B)** Comparison of the direction of two drugs intercalated in a DNA shows ActD and Dox chromophores are parallel to DNA base pairs in the current complex structure. PDB codes for each structure is given for reference.

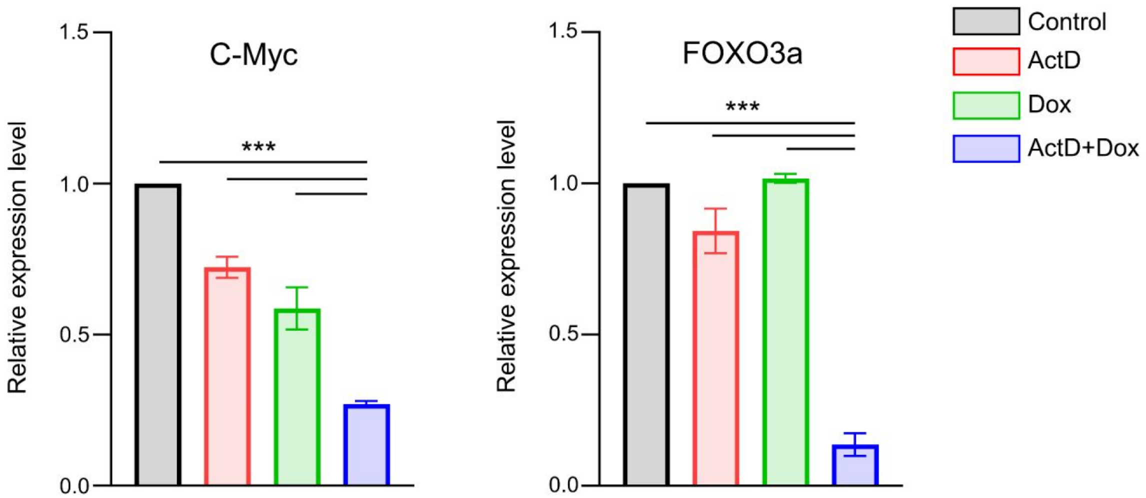

Figure S11. C-Myc and FOXO3a expression analysis upon combination treatment of ActD and Dox. RT-qPCR analysis was performed to evaluate the expression of c-Myc and FOXO3a in MDA-MB-231 cells treated with a 1:300 ratio of molar concentration of ActD and Dox for 24 h (n=3 biological replicates). Statistical significance is shown as mean ± S.D. ***$p < 0.001$.

Supplementary Tables

Table S1. List of sequences used for biophysical analysis.

Sequence ID	Oligonucleotide sequence (5'→3')
AAAA**GCCG**AAAA	5'-AAAAGCCGAAAA-3'/5'-TTTTCGGCTTTT-3'
AAA**GCACG**AAAA	5'- AAAGCACGAAAA-3'/5'-TTTTCGTGCTTT-3'
AAA**GCAACG**AAA	5'- AAAGCAACGAAA-3'/5'-TTTCGTTGCTTT-3'
AA**GCAAACG**AAA	5'- AAGCAAACGAAA-3'/5'-TTTCGTTTGCTT-3'

Table S2. IC_{50} values of five clinical chemotherapeutic drugs in MDA-MB-231, 4T1 and MCF-7 breast cancer cell lines.

Cancer cell lines	Doxorubicin (Dox)	Actinomycin D (ActD)	Cisplatin	5-Fluorouracil	Cyclophosphamide (CPX)
MDA-MB-231	1.1±0.2µM	6.5±0.9nM	37.2±5.1µM	>100 µM	>10mM
4T1	0.5±0.04µM	2.8±1.1nM	3.3±1.2µM	5.9±0.3µM	>10mM
MCF7	0.3±0.06µM	4.5±1.7nM	19.1±2.2µM	38.3±5.0µM	>10mM

Table S3. Average IC_{50} values and combination indices (CI) from different ratios of ActD and Dox treatments on breast cancer.

**ActD:Dox**	**IC_{50} (nM)**				**CI (ED_{50})**			
	1:100	1:200	1:300	1:500	1:100	1:200	1:300	1:500
MDA-MB-231	203.3	549.4	629.9	711.1	0.65	0.56	0.33	0.37
4T1	223.5	250.2	274.2	389.7	0.8	0.77	0.69	0.50
MCF-7	126.3	198.3	216.1	254.9	0.67	0.5	0.51	0.32

Table S4. Top 50 significantly downregulated DEGs in ActD+Dox treatment group.

**Ensembl_gene_id**	**Symbol**	**Description**	**Log2 fold change**
ENSG00000224057	EGFR-AS1	EGFR antisense RNA 1	-5.31
ENSG00000203392	CTD-2026K11	antisense	-5.13
ENSG00000148219	ASTN2	astrotactin 2	-4.35
ENSG00000114805	PLCH1	phospholipase C eta 1	-4.26
ENSG00000231595	AC005224	Long non-coding RNA	-4.19
ENSG00000146648	EGFR	epidermal growth factor receptor	-4.15
ENSG00000280202	RP5-1180D12	TEC	-3.95
ENSG00000172215	CXCR6	C-X-C motif chemokine receptor 6	-3.94
ENSG00000279191	RP11-803D5	TEC	-3.92
ENSG00000280543	ASAP1-IT2	ASAP1 intronic transcript 2	-3.86
ENSG00000187079	TEAD1	TEA domain transcription factor 1	-3.83
ENSG00000205810	KLRC3	killer cell lectin like receptor C3	-3.6
ENSG00000228748	RP13-39P12	antisense	-3.56
ENSG00000231887	PRH1	proline-rich protein HaeIII subfamily 1	-3.33
ENSG00000148516	ZEB1	zinc finger E-box binding homeobox 1	-3.3
ENSG00000261786	RP4-555D20	Long intervening/intergenic noncoding RNAs	-3.25
ENSG00000146950	SHROOM2	shroom family member 2	-3.23
ENSG00000260279	AC137932	antisense	-3.17
ENSG00000235944	ZNF815P	zinc finger protein 815, pseudogene	-3.16
ENSG00000167693	NXN	nucleoredoxin	-3.09
ENSG00000156299	TIAM1	T-cell lymphoma invasion and metastasis 1	-3.09
ENSG00000160191	PDE9A	phosphodiesterase 9A	-3.09
ENSG00000166446	CDYL2	chromodomain protein, Y-like 2	-3.01
ENSG00000115306	SPTBN1	spectrin beta, non-erythrocytic 1	-2.97
ENSG00000184588	PDE4B	phosphodiesterase 4B	-2.96
ENSG00000112319	EYA4	EYA transcriptional coactivator and phosphatase 4	-2.93
ENSG00000160551	TAOK1	TAO kinase 1	-2.93
ENSG00000124664	SPDEF	SAM pointed domain containing ETS transcription factor	-2.93
ENSG00000198542	ITGBL1	integrin subunit beta like 1	-2.92
ENSG00000111110	PPM1H	protein phosphatase, Mg2+/Mn2+ dependent 1H	-2.91
ENSG00000196208	GREB1	growth regulation by estrogen in breast cancer 1	-2.88
ENSG00000188039	NWD1	NACHT and WD repeat domain containing 1	-2.87
ENSG00000171365	CLCN5	chloride voltage-gated channel 5	-2.86
ENSG00000083642	PDS5B	PDS5 cohesin associated factor B	-2.86
ENSG00000125965	GDF5	growth differentiation factor 5	-2.81
ENSG00000147799	ARHGAP39	Rho GTPase activating protein 39	-2.78

ENSG00000204842	ATXN2	ataxin 2	-2.77
ENSG00000188710	QRFP	pyroglutamylated RFamide peptide	-2.75
ENSG00000102189	EEA1	early endosome antigen 1	-2.74
ENSG00000138162	TACC2	transforming acidic coiled-coil containing protein 2	-2.73
ENSG00000058056	USP13	ubiquitin specific peptidase 13 (isopeptidase T-3)	-2.71
ENSG00000169247	SH3TC2	SH3 domain and tetratricopeptide repeats 2	-2.7
ENSG00000169499	PLEKHA2	pleckstrin homology domain containing A2	-2.7
ENSG00000011566	MAP4K3	mitogen-activated protein kinase kinase kinase kinase 3	-2.69
ENSG00000102385	DRP2	dystrophin related protein 2	-2.69
ENSG00000185024	BRF1	BRF1, RNA polymerase III transcription initiation factor 90 kDa subunit	-2.66
ENSG00000134318	ROCK2	Rho associated coiled-coil containing protein kinase 2	-2.64
ENSG00000170681	MURC	muscle related coiled-coil protein	-2.63
ENSG00000120162	MOB3B	MOB kinase activator 3B	-2.63
ENSG00000154001	PPP2R5E	protein phosphatase 2 regulatory subunit B'epsilon	-2.61
TEC: To be experimentally confirmed.			

Note: Top 50 significantly downregulated DEGs sorted by log2 fold change. The list excludes DEGs from the single ActD or Dox groups, and the normalized counts' mean value is higher than 20.

Table S5. Complete blood count was performed on mice treated with the indicated high-dose treatment.

	Control	CP	CP+Dox	ActD	Dox	ActD+Dox	0.5x ActD+Dox
RBC M/uL	11.01±0.49	10.32±0.08	10.15±0.26	10.97±0.01	10.72±0.35	10.91±0.35	10.68±0.36
WBC K/uL	5.62±0.28	6.92±2.53	3.71±1.06	6.5±2.9	3.72±0.75	3.05±0.68	6.79± 0.88
NEU K/uL	0.76±0.17	1.20±0.56	0.66±0.25	0.91±0.18	0.61±0.25	0.64±0.06	1.74±0.64
LYM K/uL	3.85±0.12	5.05±1.92	2.38±0.54	5.29±2.7	2.1±0.43	2.07±0.47	4.23±1.18
MONO K/uL	0.82±0.54	0.47±0.01	0.52±0.21	0.09±0.02	0.44±0.32	0.14±0.09	0.30±0.13
EOS K/uL	0.18±0.03	0.20±0.04	0.14±0.08	0.21±0.01	0.11±0.01	0.19±0.08	0.39±0.09
BASI K/uL	0.01±0.01	0.01±0.01	0.01±0.01	0.01±0.01	0.01±0.01	0.01±0.01	0.02±0.01

Abbreviations: RBC, Red blood cells; WBC, white blood cells; NEU, Neutrophils; LYM, Lymphocytes; MONO, Monocytes; EOS, Eosinophils; BASI, Basophils.

Table S6. Crystallographic and refinement statistics of the ternary complex of ActD-Dox-d$(AGCCGT)_2$ presented in this study.

**Data collection statistics**	
Complex structure	**ActD-Dox-d$(AGCCGT)_2$**
Beamline	NSRRC, BL15A1, Taiwan
Detector type	RAYONIX MX300HE
Wavelength [Å]	1.00000
Data collection temperature [K]	100
Space group	$P6_522$
**Cell dimensions**	
a, b, c [Å]	27.00, 27.00, 205.99
α, β, γ [°]	90, 90, 120
Resolution range [Å]*	30-1.52 (1.52-1.57)
Total reflections	269500
Unique reflections	7813
Completeness [%]*	99.7 (100.0)
Mean I/σ [I]*	50.41 (12.33)
R-merge [%]*	0.070 (0.449)
Redundancy*	34.5 (38.8)
**Refinement statistics**	
R-work/R-free	0.20/0.21
No. of non-solvent atoms	379
No. of solvent atoms	93
Average B-factor [$Å^2$]	17.8
R.m.s.d. bonds lengths [Å]	0.012
R.m.s.d. bond angles [°]	2.0
**PDB code**	9JL7

*Outer shell statistics are shown in parenthesis.

Table S7. (A) DNA torsion angles, sugar puckers, (B) Local base-pair and (C) Local base-pair step parameters in the ActD-Dox-d(AGCCGT)$_2$ complex analyzed by Web3 DNA online webserver.

(A) Torsion angles and sugar pucker

		α (°)	β (°)	γ (°)	δ (°)	ε (°)	ζ (°)	χ (°)	Sugar pucker
A-DNA		-65	180	60	81	-148	-71	-160	C3'-*endo*
B-DNA		-41	-136	40	139	-133	-157	-100	C2'-*endo*
**Strand 1**	Base								
	A1	---	161.2	-172.4	161.4	-167.1	-85.0	-79.9	C3'-*exo*
	G2	-76.6	170.3	43.0	107.5	-159.9	-85.0	-90.1	O4'-*endo*
	C3	-172.0	143.1	172.6	110.2	-173.0	-104.6	-104.3	C1'-*exo*
	C4	-56.7	152.8	66.6	110.0	-158.6	-91.2	-119.6	C1'-*exo*
	G5	-55.6	-173.9	46.2	146.4	-153.0	157.6	-92.5	C2'-*endo*
	T6	94.1	-104.2	-164.1	154.6	---	---	-150.2	C2'-*endo*
**Strand 2**	A7	---	121.7	-166.5	145.4	-162.3	-91.6	-116.2	C2'-*endo*
	C8	-65.3	166.5	45.4	134.4	-115.6	176.8	-95.8	C1'-*exo*
	G9	-60.8	169.2	51.5	153.8	-168.5	-128.1	-89.9	C2'-*endo*
	G10	-60.3	179.5	41.3	136.7	-151.1	-71.1	-95.2	C2'-*endo*
	C11	168.1	139.9	-177.5	104.1	-162.0	-103.4	-114.4	O4'-*endo*
	T12	-61.3	155.9	46.8	89.3	---	---	-115.8	O4'-*endo*

(B) Local base-pair parameters

	**Shear (Å)**	**Stretch (Å)**	**Stagger (Å)**	**Buckle (°)**	**Propeller (°)**	**Opening (°)**
A-DNA	0.01	-0.18	0.02	-0.1	-11.8	0.6
B-DNA	0.00	-0.15	0.09	0.5	-11.4	0.6
**Base-pair**						
A1-T12	0.11	-0.12	-0.03	-2.86	-7.51	4.15
G2-C11	-0.38	-0.11	0.64	13.70	8.28	2.46
C3-G10	0.36	-0.18	0.31	-2.06	6.29	-0.26
C4-G9	0.12	-0.10	-0.10	13.58	-2.75	1.78
G5-C8	-0.19	-0.09	-0.34	-13.37	5.27	1.82
T6-A7	-0.17	-0.12	0.14	3.73	-4.17	1.55

(C) Local base pair step parameters

	**Shift (Å)**	**Slide (Å)**	**Rise (Å)**	**Tilt (°)**	**Roll (°)**	**Twist (°)**
A-DNA	0.00	-1.53	3.32	0.1	8.0	31.1
B-DNA	-0.02	0.23	3.32	-0.1	0.6	36.0
**Base-pair Step**						
$A_1G_2/C_{11}T_{12}$	0.59	0.59	2.90	-5.39	9.34	20.46
$G_2C_3/G_{10}C_{11}$	0.20	1.62	7.35	3.19	3.68	10.46
C_3C_4/G_9G_{10}	0.82	0.41	2.92	4.76	4.07	29.89

C_4G_5/C_8G_9	1.32	0.98	7.00	1.02	-3.83	31.58
G_5T_6/A_7C_8	-0.97	-0.14	3.05	-5.25	-5.91	27.99

Table S8. List of primer sequences used for RT-qPR experiments.

Target gene	Primer sequence
Human EGFR	Forward: 5’ -AGAAAGGCAGCCACCAAATTAGCC-3’
	Reverse: 5’- TTCCTGGCTAGTCGGTGTAAACGT-3’
Human MMP2	Forward: 5’ -CAAGGACCGGTTCATTTGGC-3’
	Reverse: 5’- ATTCCCTGCAAAGAACACAGC-3’
Human MMP9	Forward: 5’-TTGACAGCGACAAGAAGTGG-3’
	Reverse: 5’- GCCATTCACGTCGTCCTTAT-3’
Human c-MYC	Forward: 5’-CCTGGTGCTCCATGAGGAGAC-3’
	Reverse: 5’-CAGACTCTGACCTTTTGCCAGG-3’
Human FOXO3a	Forward: 5’-TCTACGAGTGGATGGTGCGTTG-3’
	Reverse: 5’-CTCTTGCCAGTTCCCTCATTCTG-3’
Human GAPDH	Forward: 5’-GGCATCCTGGGCTACACTGA-3’
	Reverse: 5’-GGAGTGGGTGTCGCTGTTG-3’

Table S9. List of primary and secondary antibodies for western blot.

**Primary antibody**				
**Target**	**Catalog No.**	**Host**	**Dilution**	**Manufacture**
EGFR	GTX35199	Rabbit	1:2000	Gene Tex
Phospho-ERK1/2	9101	Rabbit	1:1000	Cell signaling
ERK1/2	A4782	Rabbit	1:1000	ABclonal
Phospho-AKT1	AP0004	Rabbit	1:1000	ABclonal
Akt1	A22533	Rabbit	1:1000	ABclonal
MMP2	A6247	Rabbit	1:1000	ABclonal
MMP9	A0289	Rabbit	1:1000	ABclonal
Vimentin	A0326	Rabbit	1:1000	ABclonal
EpCAM	A19301	Rabbit	1:1000	ABclonal
GAPDH	A19056	Rabbit	1:2000	ABclonal
**Secondary antibody**				
Goat anti-Rabbit IgG (H+L), HRP	G-21234	Goat	1:5000	Invitrogen
